# Synergistic activity of combined inhibition of anti-apoptotic molecules in B-cell precursor ALL

**DOI:** 10.1038/s41375-021-01502-z

**Published:** 2022-01-14

**Authors:** Felix Seyfried, Felix Uli Stirnweiß, Alexandra Niedermayer, Stefanie Enzenmüller, Rebecca Louise Hörl, Vera Münch, Stefan Köhrer, Klaus-Michael Debatin, Lüder Hinrich Meyer

**Affiliations:** 1 Department of Pediatrics and Adolescent Medicine, Ulm University Medical Center, Ulm, Germany; 2 International Graduate School in Molecular Medicine, Ulm University, Ulm, Germany; 3 St. Anna Children’s Hospital, Department of Pediatric Hematology and Oncology, Vienna, Austria

**Keywords:** Targeted therapies, Cancer therapeutic resistance

## Abstract

Targeting BCL-2, a key regulator of survival in B-cell malignancies including precursor B-cell acute lymphoblastic leukemia, has become a promising treatment strategy. However, given the redundancy of anti-apoptotic BCL-2 family proteins (BCL-2, BCL-XL, MCL-1), single targeting may not be sufficient. When analyzing the effects of BH3-mimetics selectively targeting BCL-XL and MCL-1 alone or in combination with the BCL-2 inhibitor venetoclax, heterogeneous sensitivity to either of these inhibitors was found in ALL cell lines and in patient-derived xenografts. Interestingly, some venetoclax-resistant leukemias were sensitive to the MCL-1-selective antagonist S63845 and/or BCL-XL-selective A-1331852 suggesting functional mutual substitution. Consequently, co-inhibition of BCL-2 and MCL-1 or BCL-XL resulted in synergistic apoptosis induction. Functional analysis by BH3-profiling and analysis of protein complexes revealed that venetoclax-treated ALL cells are dependent on MCL-1 and BCL-XL, indicating that MCL-1 or BCL-XL provide an Achilles heel in BCL-2-inhibited cells. The effect of combining BCL-2 and MCL-1 inhibition by venetoclax and S63845 was evaluated in vivo and strongly enhanced anti-leukemia activity was found in a pre-clinical patient-derived xenograft model. Our study offers in-depth molecular analysis of mutual substitution of BCL-2 family proteins in acute lymphoblastic leukemia and provides targets for combination treatment in vivo and in ongoing clinical studies.

## Introduction

B-cell precursor acute lymphoblastic leukemia (BCP-ALL) cells are characterized by an imbalance of cell death and survival pathways. The intrinsic apoptosis pathway is controlled at the mitochondria by the interplay of pro- and anti-apoptotic proteins of the B-cell lymphoma 2 (BCL-2) family [1]. These proteins, presenting at least one BCL-2 homology (BH) domain, are crucial for the decision of the cell fate between survival and cell death [2]. The multidomain anti-apoptotic BCL-2 family proteins BCL-2, BCL-XL, BCL-W, MCL-1 and BFL1 prevent apoptosis by counteracting BH3-only proteins or by binding to BAX and BAK [3]. Pro-apoptotic sensitizer BH3-only proteins including BAD, BIK, NOXA and others bind to and antagonize anti-apoptotic BCL-2 family proteins. Activator proteins, such as BIM and BID, carry out their function by directly activating BAX and BAK [4]. Once activated, BAX and BAK form oligomers leading to pore formation and mitochondrial outer membrane permeabilization (MOMP) followed by release of cytochrome c and other pro-death proteins from mitochondria, ultimately resulting in activation of caspases and cell death [5].

Evading apoptosis is a hallmark of human cancer cells [6] and often promoted by the activation of anti-apoptotic proteins, particularly in BCP-ALL [7–9]. BH3-mimetics have been developed to initiate apoptosis signaling by inhibiting the anti-apoptotic activity of selected BCL-2 family proteins [10]. The BCL-2 selective inhibitor venetoclax (ABT-199) has shown efficacy in different hematological malignancies such as chronic lymphocytic leukemia (CLL) [11], acute myeloid leukemia (AML) [12] and T-cell acute lymphoblastic leukemia [13]. In BCP-ALL, heterogeneous responses have been observed and the mitochondrial dependence on BCL-2 is a marker of response for the anti-leukemia activity of venetoclax [14–17]. However, other anti-apoptotic members of the BCL-2 family proteins, including MCL-1 and BCL-XL may contribute to cell survival in BCP-ALL counteracting venetoclax sensitivity leading to poor treatment response. In this context, inhibitors targeting MCL-1 or BCL-XL might overcome venetoclax insensitivity and are therefore of high interest as treatment options for various malignancies, in particular in combination with other therapies including venetoclax [18].

In this study, we systematically investigated the effects of venetoclax (BCL-2), S63845 (MCL-1), and A-1331852 (BCL-XL) side-by-side in BCP-ALL, identifying heterogeneous sensitivities of all three inhibitors. We identified a profound switch in the functional dependence from BCL-2 to MCL-1 and BCL-XL in ALL cells upon exposure to venetoclax. Attacking this mutual interplay by co-inhibition of BCL-2 with MCL-1 demonstrated strong synergistic activity in primary patient-derived xenograft (PDX) samples ex vivo and in vivo.

## Materials and methods

### BCP-ALL cell lines

RS4;11, KOPN-8, REH, EU-3, RCH-ACV, and NALM-6 cells were purchased (DSMZ, Germany), UoCB6 cells were kindly provided by J. Rowley (USA) and cultured (RPMI-1640 medium, 20% fetal bovine serum, 1% L-Glutamine, 1% Penicillin/Streptomycin; 5% CO_2_, 37 °C).

### BCP-ALL patient-derived xenograft samples

Primary leukemia samples of BCP-ALL patients were collected after written informed consent in accordance with the institution’s ethical review board. Patient-derived xenograft samples were generated by intravenous transplantation of ALL cells into female NOD/SCID mice (NOD.CB17-Prkdcscid, Charles River) as described [19]. Animal experiments were approved (Regierungspräsidium Tübingen, Tierversuch Nr. 1260).

### Cell viability assays

To assess half maximal effective concentration (EC_50_) values, cells were exposed to venetoclax, S63845 and A-1331852 and cell death was analyzed either according to propidium iodide (PI) positivity (cell lines) or forward/side scatter criteria (PDX samples). Comparison of both methods yielded similar results of cell death detection as shown for RS4;11 (Supplementary Fig. 1). Combination effects were analyzed by dose-response matrix analyses upon exposure to inhibitors (2.5, 5, 25, 50, 250, 500, and 2500 nM) for 48 h in cell lines and for 24 h in PDX samples, followed by cell death determination (PI staining). Apoptosis of ALL cells was assessed by staining with Annexin V (APC, #550475, BD Pharmingen) and PI.

### Dynamic BH3 profiling

Dynamic BH3 profiling was performed as described [20, 21]: Cells were exposed to venetoclax (RS4;11, KOPN-8: 2 h; all others: 4 h), permeabilized (digitonin), and exposed to BH3-peptides. Cytochrome c release was analyzed (staining with Dapi and anti-cytochrome c antibody, Attune NxT Flow Cytometer). Cytochrome c median fluorescence intensities (MFI) were determined and normalized to MFIs of negative (DMSO) and positive (Alamethicin) controls.

### Intracellular protein staining

Cells were intracellularly stained with anti-BCL-2, anti-BCL-XL, anti-MCL-1, IgG1 and IgG isotype antibodies in triplicates and analyzed by flow cytometry. MFIs were normalized to respective isotype controls.

### Immunoprecipitation and immunoblotting

For immunoprecipitation, protein lysates were incubated with 88 ng of BIM Rabbit mAb (overnight, 4 °C) followed by Protein A Agarose beads (2 h, 4 °C). The beads were washed 5x with lysis buffer and the precipitates were then subjected to western blot analyses. For immunoblotting, anti-BCL-2, anti-BCL-XL, anti-MCL-1, anti-BIM, and anti-alpha-Tubulin antibodies were used as primary antibodies and mouse anti-rabbit IgG-HRP, mouse IgGκ BP-HRP or goat anti-mouse IgG_1_-HRP as secondary antibodies. Immunoblots were developed using chemiluminescence.

### In vivo treatment

Upon leukemia engraftment (5% human ALL cells in peripheral blood), mice were treated with venetoclax (25 mg/kg/d orally), S63845 (25 mg/kg/d intraperitoneally), the combination or with vehicle (two weeks days 1-5).

### Statistical analysis

Statistical analyses were performed with GraphPad Prism 9 software and Microsoft Excel. 3D scatter plots of EC_50_ values were generated using Plotly software [22]. Combination effects and synergy scores were analyzed (Synergyfinder) using the Bliss independence model [23–25].

Further details on the methods used can be found in the supplementary information.

## Results

### BCL-2, BCL-XL, and MCL-1 are attractive therapeutic targets in B-cell precursor acute lymphoblastic leukemia

**Fig. 1:**
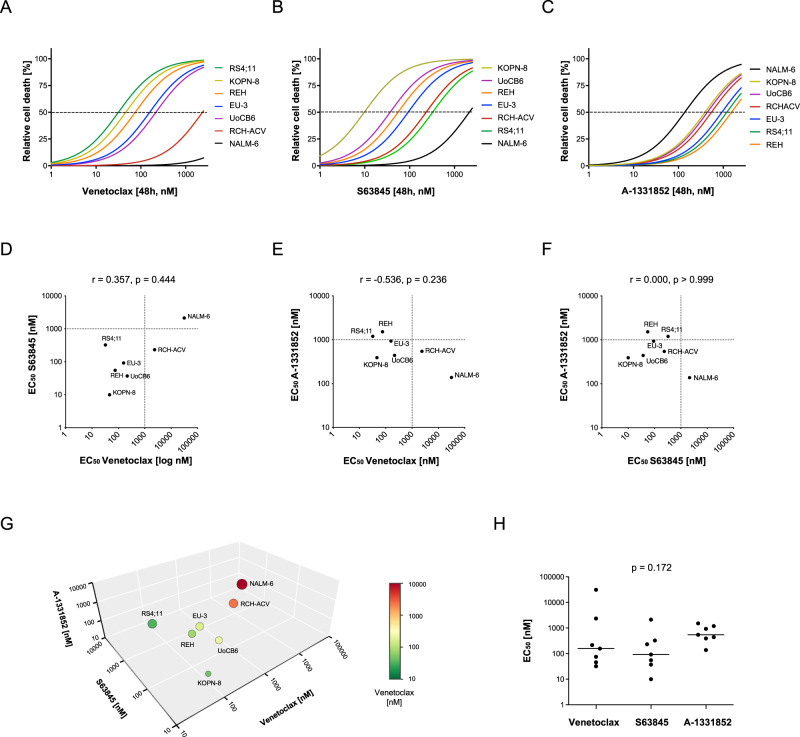
BCP-ALL cell lines present heterogeneous sensitivity to cell death induced by BH3-mimetics. BCP-ALL cell lines were exposed for 48 hours to increasing concentrations (2.5, 5, 25, 50, 250, 500, and 2500 nM) of (**A**) venetoclax, (**B**) S63845 or (**C**) A-1331852 before analysis of cell death by propidium iodide staining and flowcytometry. *N* = 3 independent experiments in triplicates. **D**–**F** Association of the EC_50_ values of venetoclax, S63845 and A-1331852 of all cell lines. Spearman correlation; r, correlation coefficient; p, significance. (**G**) 3D scatter plot of the EC_50_ values of the three inhibitors. The colors of the symbols represent the half maximal effective concentration (EC_50_) values of venetoclax as indicated in the legend and the sizes indicate the EC_50_ values of S63845. **H** Scatter plot of EC_50_ values of the three BH3-mimetics with individual data points of single cell lines and medians (lines). Kruskal-Wallis test; p, significance.

To determine the capability of selected anti-apoptotic BCL-2 family members as therapeutic targets in BCP-ALL, we used inhibitors selectively antagonizing BCL-2 (venetoclax), MCL-1 (S63845), and BCL-XL (A-1331852). We analyzed cell death rates (propidium iodide positivity) in seven BCP-ALL cell lines upon exposure to increasing concentrations of the three antagonists. Varying response rates with EC_50_ values from nanomolar to micromolar concentrations were found for all inhibitors in the different cell lines (Fig. 1A–C, Supplementary Fig. 2). For example, RCH-ACV and NALM-6 showed both insensitivity to venetoclax and NALM-6 also to S63845 with EC_50_ values above 1 µM, whereas the other lines showed lower EC_50_ values, indicating sensitivity to BCL-2 and MCL-1 inhibition. In contrast, only NALM-6 showed sensitivity for A-1331852, while the other lines showed higher EC_50_ values. No clear association was found between sensitivities to the three inhibitors (Fig. 1D–G). Comparing the overall efficacy of the three compounds analyzing the EC_50_ values of cell lines did not result in a significant difference (Fig. 1H). Interestingly, leukemia characteristics of the cell lines were not found to be associated with inhibitor sensitivities (Supplementary Fig. 3).

Taken together, varying sensitivities of BCP-ALL cell lines were found for all three inhibitors with a trend towards higher sensitivity for venetoclax and S63845 as compared to A-1331852.

### S63845 and A-1331852 provide potential alternative treatment options for selected venetoclax insensitive leukemias

**Fig. 2:**
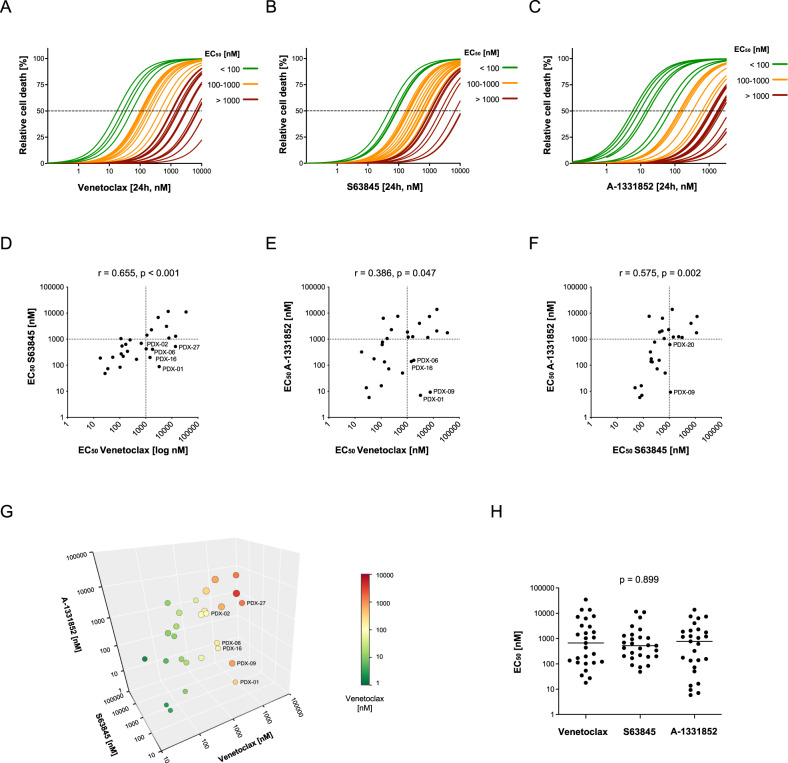
Side-by-side analysis of venetoclax, S63845, and A-1331852 in BCP-ALL xenograft samples. Cell death rates were assessed by flowcytometry according to forward and side scatter criteria following exposure of primary BCP-ALL xenograft samples for 24 h to triplicates of increasing concentrations (1, 5, 10, 50, 100, 250, 500 nM, 1, 5 and 10 µM) of (**A**) venetoclax, (**B**) S63845 or (**C**) A-1331852. The EC_50_ curves are color-coded as indicated in the legend. **D**–**F** Association of the EC_50_ values of venetoclax, S63845, and A-1331852. Spearman correlation; r, correlation coefficient; p, significance. **G** 3D scatter plot showing an association of the EC_50_ values of venetoclax, S63845, and A-1331852. Samples which are insensitive to venetoclax (EC_50_ > 1 µM) but that are sensitive to at least one of the other two inhibitors are labeled in the graph. The colors of the symbols represent the EC_50_ values of venetoclax as indicated in the legend and the sizes indicate the EC_50_ values of S63845. **H** Scatter plot of the EC_50_ values of the three inhibitors with individual data points of single PDX samples and medians (lines). Kruskal-Wallis test; p, significance.

As cell lines do not sufficiently reflect the characteristics of primary BCP-ALL, we next investigated the anti-leukemic effects of the three inhibitors in a cohort of 27 PDX samples. As observed for the cell lines, the different BH3-mimetics showed heterogeneous activities in the individual primary PDX samples (Fig. 2A–C, Supplementary Fig. 4). EC_50_ values below 1 µM were observed for all three inhibitors in about one half of the PDX samples, indicating sensitivity (EC_50_ < 1 µM) in 14/27 (52%) of PDX samples for venetoclax; 18/27 (67%) for S63845 and 14/27 (52%) for A-1331852. Within the PDX samples, sensitivities to all three BH3-mimetics were associated with each other (Fig. 2D–G). Importantly, some samples with insensitivity to one inhibitor showed EC_50_ values below 1 µM indicating sensitivity to another BH3-mimetic. Venetoclax insensitive (EC_50_ > 1 µM) leukemias showed sensitivity to at least one of the other two inhibitors in six out of 13 cases: two out of 13 venetoclax resistant samples were only sensitive to S63845 (PDX-02 and -27), one sample was exclusively sensitive to A-1331852 (PDX-09) and three samples were sensitive to both inhibitors (PDX-16, -06, and -01) (Fig. 2D, E, G). Despite variable responses, the EC_50_ values did not differ significantly between the inhibitors (Fig. 2H).

BCP-ALL is a heterogeneous disease with different subgroups characterized by expression of surface markers and recurrent genetic aberrations. In order to investigate whether specific ALL subgroups are associated with sensitivity to a specific inhibitor, we summarized the different characteristics sorting the individual PDX samples according to increasing EC_50_ values of each inhibitor (Supplementary Fig. 5A–C). No specific subgroup was found to be particularly sensitive to one of the inhibitors. Some leukemias showed insensitivity to all three inhibitors (*n* = 7, EC_50_ > 1000 nM, Supplementary Fig. 4) but did not share common characteristics (Supplementary Fig. 5D).

We next examined the protein expression levels of the target molecules of the three inhibitors in BCP-ALL PDX samples using intracellular staining and flowcytometry (Supplementary Fig. 6A, B). We found an association of venetoclax sensitivity (low EC_50_ values) with high BCL-2 protein expression and with low MCL-1 levels (Table 1). In contrast, we did not find a clear association of target protein levels with sensitivity to MCL-1 or BCL-XL antagonism. In addition, we assessed expression levels of the pro-apoptotic molecules BIM, BID, BAX, BAK, Noxa, BAD, PUMA, and BMF in the BCP-ALL cell lines by western blot. No overall association of expression of pro-apoptotic proteins with sensitivities to the three inhibitors was observed (Supplementary Fig. 7A, B). Thus, responses to BH3-mimetics are not reflected by basal levels of single molecules but rather mediated by the functional interplay of the different regulators.

**Table 1:** Association of EC_50_ values of BH3-mimetics with protein levels.

		BCL-2	BCL-XL	MCL-1
Venetoclax	r_s_	–0.480	0.171	0.530
	p	0.018*	0.424	0.008*
S63845	r_s_	–0.120	0.117	0.357
	p	0.577	0.585	0.087
A-1331852	r_s_	0.174	–0.263	0.034
	p	0.418	0.214	0.873

Association of EC_50_ values of BH3-mimetics with protein levels of target molecules. Patient-derived xenograft BCP-ALL (*N* = 24). Spearman correlation; r, correlation coefficient; *p*, significance; * *p* ≤ 0.05.

### Dynamic BH3 profiling shows increased dependence on MCL-1 and BCL-XL in the presence of venetoclax

**Fig. 3:**
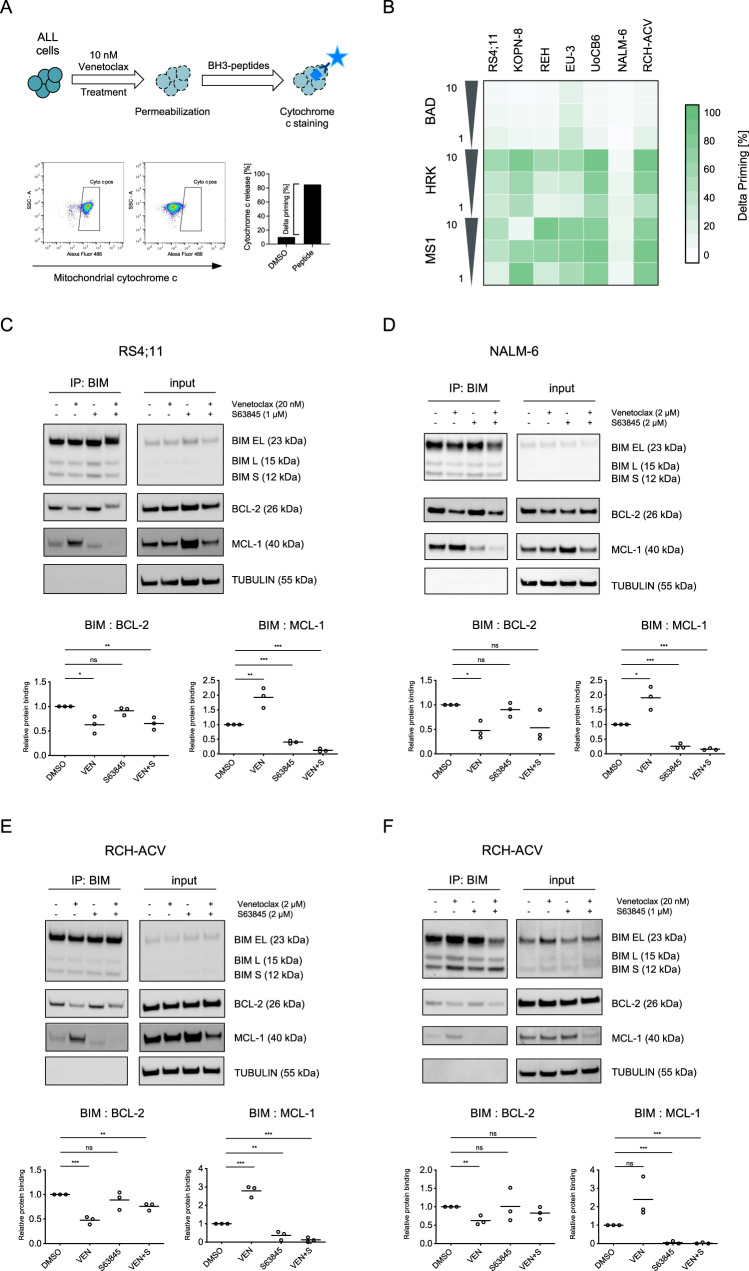
BCL-XL and MCL-1 mediate resistance of ALL cells to venetoclax. The interaction of proteins of the BCL-2 family was investigated in BCP-ALL cell lines upon exposure to BH3-mimetics. **A** Graphical schematic of dynamic BH3 profiling (DBP) analyzing BCP-ALL cell lines. **B** Cells were exposed to 10 nM venetoclax for 2 h (RS4;11, KOPN-8) or 4 h (all others) followed by permeabilization and incubation with the pro-apoptotic BH3-peptides BAD (indicating BCL-2 dependence), HRK (BCL-XL) and MS1 (MCL-1). Cells were then fixed and stained with an anti-cytochrome c antibody binding exclusively to mitochondrial cytochrome c. Delta priming was calculated as follows: Delta priming (%) = venetoclax-induced cytochrome c release (%) – DMSO control-induced cytochrome c release (%). The heatmap of DBP results shows increased mitochondrial priming in cell lines upon venetoclax-exposure to the MS1 peptide (MCL-1 dependence) and to HRK (BCL-XL). Immunoprecipitation (IP) analysis of BIM and detection of co-precipitated/BIM-bound BCL-2 and MCL-1 upon exposure to venetoclax, S63845 or the combination of both inhibitors for 4 h. **C** RS4;11, venetoclax sensitive; exposure to 20 nM venetoclax, 1 µM S63845. **D** NALM-6, venetoclax insensitive; 2 µM venetoclax, 2 µM S63845. **E** RCH-ACV, venetoclax insensitive; 2 µM venetoclax, 2 µM S63845 and (**F**) RCH-ACV, *low* concentrations 20 nM venetoclax, 1 µM S63845. The immunoprecipitation lanes show the interaction of BIM with BCL-2 and MCL-1 and the input lanes show the whole protein lysates. Diagrams show densitometric quantification of co-precipitated BCL-2 (BIM: BCL-2) or MCL-1 (BIM: MCL-1) in the respective condition relative to no inhibitor control summarizing three independent experiments (corresponding replicates see Supplementary Fig. 8). Unpaired two-tailed Student’s *t* test; significance ***, *p* < 0.001; **, *p* < 0.01; *, *p* < 0.05; ns, not significant.

To investigate whether MCL-1 and BCL-XL mediate resistance to apoptosis induction by venetoclax, we used dynamic BH3 profiling analyzing dependencies of BCP-ALL cells on different anti-apoptotic proteins in response to treatment with a specific drug [21], in our case venetoclax. Upon drug exposure, synthetic pro-apoptotic BH3-peptides were used to detect the impact of the main anti-apoptotic BCL-2 family proteins for cell survival: recombinant BAD for BCL-2/BCL-XL and BCL-W dependence, HRK for BCL-XL and MS1 for MCL-1 [26, 27]. Mitochondrial cytochrome c release was then measured as an early event of apoptosis signaling. The amount of change in cytochrome c release between treatment-naïve and treated leukemia cells (delta priming) indicates a shift in their dependence on anti-apoptotic proteins (Fig. 3A). We found that exposure of BCP-ALL cell lines to venetoclax at low nanomolar concentrations resulted in enhanced mitochondrial cytochrome c release by HRK and MS1 (median delta priming 27% for HRK and 75% for MS1), demonstrating a switch from BCL-2 dependence to combined dependence on BCL-XL and MCL-1 in the presence of venetoclax (Fig. 3B). Thus, upon BCL-2 antagonism by venetoclax the suppressed pro-survival signals of BCL-2 are substituted by BCL-XL and MCL-1.

### Shuttling of BIM from BCL-2 to MCL-1 and vice versa upon venetoclax and S63845 can be blocked by co-inhibition of BCL-2 and MCL-1

To further elucidate these mechanisms of varying functional dependencies of ALL cells on anti-apoptotic apoptosis regulators, we studied the interaction of BCL-2 family proteins with BIM, the most important pro-apoptotic activator of BAX [28] which also showed high expression in BCP-ALL cells (Supplementary Fig. 7A). Immunoprecipitation of BIM and co-precipitation of BCL-2 or MCL-1 was analyzed to study binding and release of BIM from BCL-2 or MCL-1 upon exposure to venetoclax, S63845 or the combination of both inhibitors. First, we investigated a venetoclax-sensitive (RS4;11) and an insensitive ALL cell line (NALM-6) using low or high venetoclax/S63845 concentrations corresponding to their inhibitor sensitivities. A clear displacement of BIM from BCL-2 and significantly increased binding to MCL-1 (RS4;11, *P* < 0.01; NALM-6, *P* < 0.05) was observed upon venetoclax exposure, while binding of BIM to MCL-1 was significantly disrupted upon exposure to S63845 (both lines *P* < 0.001) along with compensational binding to BCL-2 (Fig. 3C, D, Supplementary Fig. 8A, B). Moreover, we performed the same binding analysis in an additional venetoclax insensitive line (RCH-ACV). Using high inhibitor concentrations, a significant compensatory binding of BIM to MCL-1 upon venetoclax (*P* < 0.001) was found (Fig. 3E, Supplementary Fig. 8C). In contrast, using lower inhibitor concentrations in the same line RCH-ACV, an overall reduced BIM shuttling was observed (Fig. 3F, Supplementary Fig. 8D). Importantly, combining venetoclax and S63845 reduced binding to both, BCL-2 and MCL-1, indicating prevention of alternative binding and increased release of BIM, ultimately leading to apoptosis induction. Interestingly, upon BCL-2 (venetoclax) and BCL-XL (A-1331852) inhibition a similar mechanism of alternate BIM binding to BCL-2 or BCL-XL and increased BIM release upon inhibitor combination was observed (Supplementary Fig. 9). Release of apoptosis activating BIM from BCL-2 and capture by anti-apoptotic molecules like MCL-1 is a rapid process occurring within 1 h after venetoclax exposure, as shown by a time-course precipitation analysis (Supplementary Fig. 10).

Thus, given the association of high BCL-2 and low MCL-1 levels with venetoclax sensitivity (Table 1) and the identified mechanism of alternative binding of BIM to BCL-2 or MCL-1, this indicates that the binding capacities of the different regulators determine the amount of free pro-apoptotic molecules and thereby regulate apoptosis induction and venetoclax sensitivity.

### Genetic depletion of MCL-1 increases sensitivity to venetoclax in ALL cell lines

**Fig. 4:**
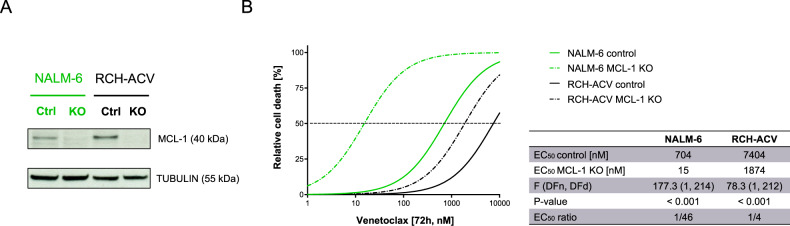
Genetic depletion of MCL-1 sensitizes ALL cells to venetoclax. Knockout of MCL-1 was performed using CRISPR/Cas9 gene editing in NALM-6 and RCH-ACV. **A** Successful knockout of MCL-1 was confirmed by western blot. **B** MCL-1 knockout cells and their corresponding controls were exposed to increasing concentrations of venetoclax followed by assessment of cell death by flowcytometry by forward/side scatter criteria in order to determine half maximal effective concentrations. *N* = 3 independent experiments in triplicates. Differences of MCL-1 knockout cell lines and corresponding control cells were determined by extra-sum-of-squares F test. F value; degrees of freedom numerator (DFn) and denominator (DFd); p, significance.

To further interrogate the role of MCL-1 for the susceptibility of ALL cells to cell death induction by venetoclax, we utilized a genetic approach for the depletion of MCL-1 using the CRISPR/Cas9 system. Depletion of MCL-1 was carried out in NALM-6 and RCH-ACV cells, which have both shown venetoclax insensitivity presenting EC_50_ values above 1 µM (Fig. 1A). The MCL-1 knockout was confirmed by western blot analysis (Fig. 4A). Upon MCL-1 knockout, we found a significant 4-fold increase in venetoclax sensitivity in RCH-ACV cells (*P* < 0.001) and an increase of even 46-fold in NALM-6 (*P* < 0.001; Fig. 4B). Thus, different extents of sensitization were observed in both cell lines, suggesting that other mechanisms will further affect venetoclax sensitivity. However, the effective sensitization of venetoclax resistant cell lines by genetic MCL-1 depletion provides a strong rationale for the combined inhibition of MCL-1 and BCL-2.

### Combined targeting of BCL-2 with MCL-1 or BCL-XL results in synergistic cell death induction

**Fig. 5:**
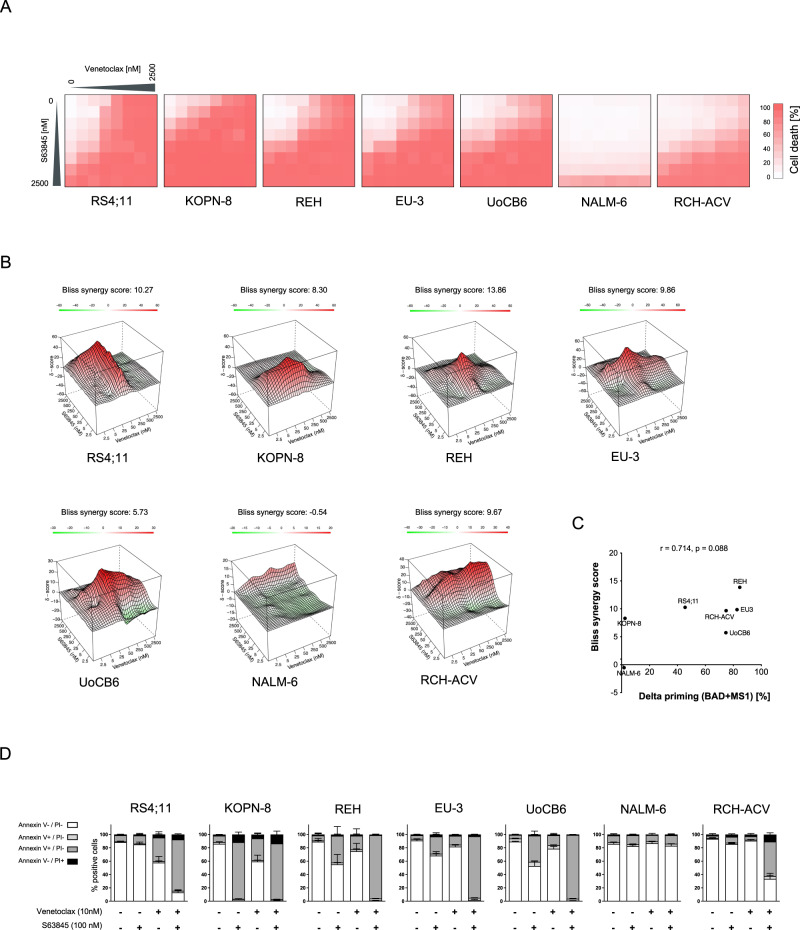
Synergistic cell death induction by venetoclax and S63845 in BCP-ALL cell lines. BCP-ALL cell lines were exposed to increasing concentrations of venetoclax, S63845 or combinations of both inhibitors. **A** Cell death rates were analyzed by flowcytometry after 48 h by propidium iodide (PI) staining. The heatmaps show cell death rates of dose-response matrix analyses of the cell lines indicated. *N* = 3 independent experiments in triplicates. **B** Interaction landscapes of the dose-response matrix analyses are shown. To estimate synergy, δ-scores were calculated using synergyfinder. Synergistic effects are shown in red, additive effects in white and antagonistic effects in green. The shown Bliss synergy score indicates the average synergy score over the dose-response matrix. **C** Association of the Bliss synergy scores of all cell lines with delta priming response values to BAD + MS1 reflecting combined dependence on BCL-2 and MCL-1. Spearman correlation; r, correlation coefficient; p, significance. **D** Annexin V and PI staining was assessed upon exposure of cells for 48 h to 10 nM venetoclax and/or 100 nM S63845 to determine apoptotic cells. The fractions of viable cells (Annexin V-/PI-), early apoptotic cells (Annexin V + /PI-), late apoptotic cells (Annexin V + /PI + ) and necrotic cells (Annexin V-/PI + ) are shown. *N* = 3 independent experiments in triplicates.

As genetic MCL-1 depletion resulted in a clear sensitization of ALL cells to cell death induced by venetoclax, we next analyzed the activity of venetoclax together with the MCL-1 inhibitor S63845. BCP-ALL cell lines were exposed to increasing venetoclax and S63845 doses (multi-dose matrix combining seven concentrations of each inhibitor) estimating cell death rates (PI staining, Fig. 5A) and determining synergy scores (Bliss independence model [23, 24]; Fig. 5B). Enhanced cell death rates were found in all cell lines upon combination of venetoclax and S63845 as compared to the inhibitors alone, however to a variable extent with almost no cell death induction in NALM-6 cells, which also were insensitive to both inhibitors alone (Fig. 1, Supplementary Fig. 2). Accordingly, synergistic activities were observed upon concomitant inhibition of BCL-2 and MCL-1 in all lines but NALM-6, including the venetoclax resistant line RCH-ACV (Figs. 1, 5B, Supplementary Fig. 2). Interestingly, high synergistic activity (high Bliss synergy score) is associated with increased dependence on BCL-2 and MCL-1 (high cell death priming upon BAD and MS1 exposure compared to control) (Fig. 5C). Annexin/PI staining confirmed induction of apoptosis in BCP-ALL cells upon inhibitor exposure (Fig. 5D, Supplementary Fig. 11).

**Fig. 6:**
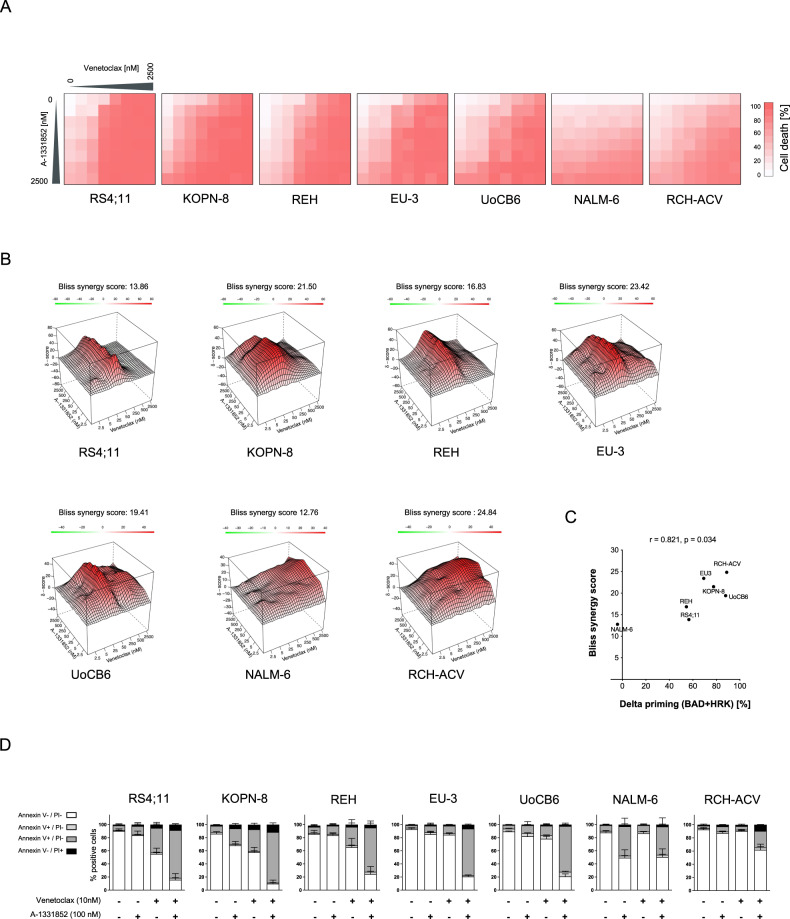
Synergistic cell death induction by venetoclax and A-1331852 in BCP-ALL cell lines. Cell death was assessed upon exposure of BCP-ALL cell lines to increasing concentrations of venetoclax and/or A-1331852 for 48 h. **A** The heatmaps show dose-response matrix analyses based on cell death assessed by propidium iodide (PI) staining of the cell lines indicated. *N* = 3 independent experiments in triplicates. **B** Interaction landscapes of the combination effects are shown. δ-scores were calculated using synergyfinder. The color encodes the δ-score (red synergistic, white additive effect and green antagonistic) and the Bliss synergy scores shown indicate the average score over the dose-response matrix. **C** Association of the calculated Bliss synergy scores with the delta priming responses to BAD + HRK (reflecting combined dependence on BCL-2 and BCL-XL) in all cell lines. Spearman correlation; r, correlation coefficient; p, significance. **D** Annexin V and PI staining following exposure to 10 nM venetoclax and/or 100 nM A-1331852 for 48 hours. Quantification of viable cells (Annexin V-/PI-), early apoptotic cells (Annexin V + /PI-), late apoptotic cells (Annexin V + /PI + ) and necrotic cells (Annexin V-/PI + ) is shown. *N* = 3 independent experiments in triplicates.

As dynamic BH3 profiling demonstrated an increased dependence of venetoclax treated ALL cells not only on MCL-1 but also on BCL-XL, we also investigated the combination of venetoclax and A-1331852. Clearly enhanced cell death and synergistic activity was observed in all lines upon co-treatment with both inhibitors (Fig. 6A, B). In addition, high synergistic activity of BCL-2 and BCL-XL inhibition is associated with high BCL-2 and BCL-XL dependence seen in BH3-profiling (cell death priming by BAD and HRK peptides) (Fig. 6C). Moreover, concomitant inhibition of BCL-2 and BCL-XL led to apoptosis induction (Fig. 6D). Thus, although venetoclax was more active than A-1331852 in inducing cell death in the majority of cell lines (Fig. 1), these cell lines were sensitized to venetoclax by simultaneous exposure to nanomolar concentrations of A-1331852.

### Synergistic activity of combined inhibition of anti-apoptotic molecules in patient-derived xenograft ALL

**Fig. 7:**
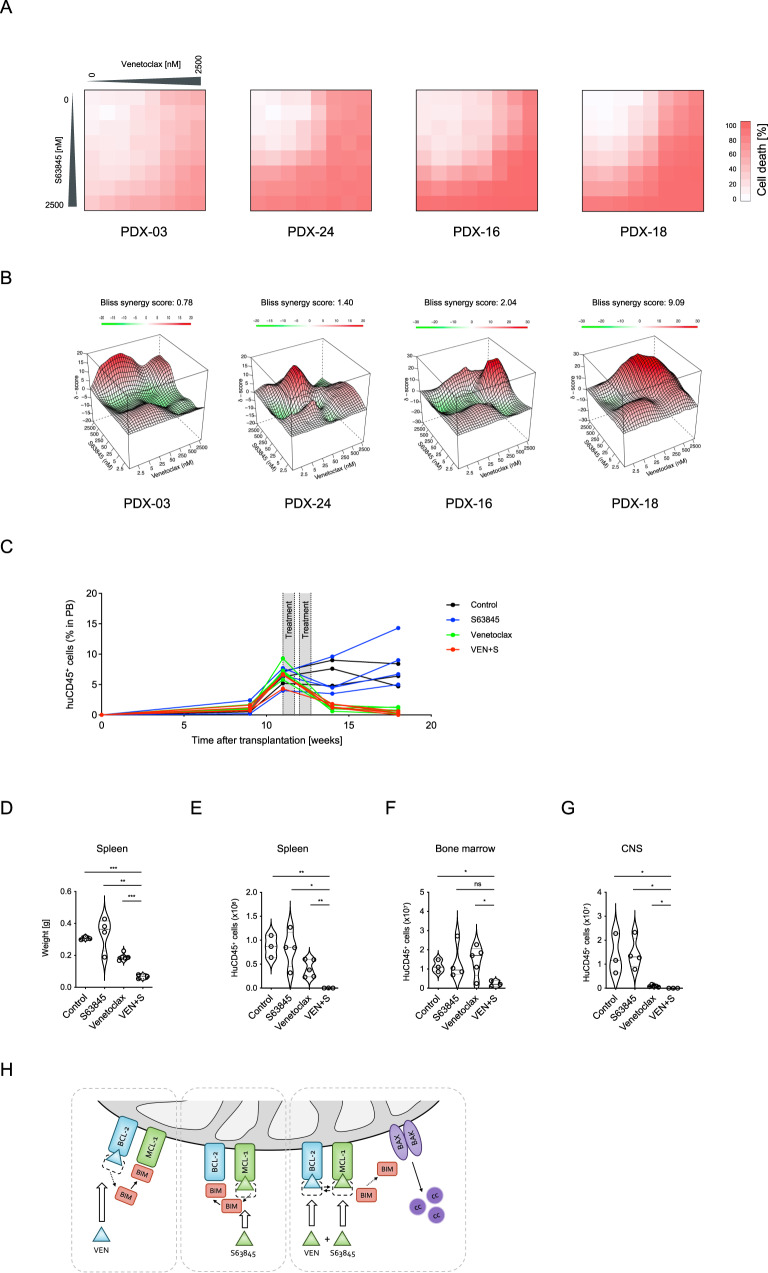
Synergistic activity of venetoclax and S63845 in patient-derived xenograft ALL. Combination effects of venetoclax and S63845 were evaluated in primary BCP-ALL xenograft samples. **A** Cell death rates of patient-derived xenograft ALL cells were analyzed by propidium iodide staining following exposure of cells to venetoclax and/or S63845 at increasing concentrations for 24 h in triplicates. **B** Synergyfinder was used to visualize combination effects (red indicating synergism, white additive effect and green antagonism) and to calculate δ-scores based on the Bliss independence model. The average synergy scores over the dose-response matrix for each sample are shown. **C** Experimental treatment schematic and human CD45^+^ (huCD45^+^) cells in mouse peripheral blood. NOD/SCID mice were transplanted with a high-risk leukemia (PDX-18) derived from an infant ALL and treatment was started upon manifestation of ≥ 5% human leukemia cells in the peripheral blood (huCD45^+^). Engrafted mice (*N* = 3–5 per group) were treated with vehicle, venetoclax (25 mg/kg per day), S63845 (25 mg/kg per day) or the combination of both compounds for five days per week for two consecutive weeks as indicated in the scheme. Leukemia burden was analyzed by (**D**) spleen weight and (**E**) absolute huCD45^+^ cell count in spleen, (**F**) bone marrow and (**G**) central nervous system of the mice. Violin plots show individual data points of single mice and median (solid) and quartile (dotted) lines for each group. Unpaired two-tailed Student’s *t* test was used to calculate *p* values. **H** When ALL cells are exposed to venetoclax (VEN), pro-apoptotic BIM is displaced from BCL-2 to MCL-1. Conversely, increased BCL-2/BIM binding is present upon exposure to S63845. Combined BCL-2 and MCL-1 blocking proficiently induces release of the apoptosis activator BIM leading to activation of BAX and BAK and triggering of downstream apoptosis signaling.

Based on our findings of increased dependence on MCL-1 or BCL-XL, sequestration of the apoptosis activator BIM upon venetoclax treatment and synergistic activity in BCP-ALL cell lines, we extended our analyses and evaluated anti-leukemia activity of combined BCL-2 and MCL-1 or BCL-XL inhibition in primary patient-derived xenograft ALL samples. Using increasing doses of venetoclax, S63845 or A-1331852 and their combinations in a multi-dose matrix titration, four PDX ALL samples were analyzed. Like in ALL cell lines, BCL-XL or MCL-1 inhibition increased the sensitivity to venetoclax induced cell death showing synergistic activity in all xenografts (Fig. 7A, B; Supplementary Fig. 12).

Finally, based on the synergistic activity of concomitant inhibition of anti-apoptotic regulators, we evaluated the combinatorial activity of venetoclax and S63845 in vivo. A high-risk, *KMT2A-MLLT1* positive, infant, pro-B ALL (PDX-18) was used and transplanted onto NOD/SCID mice. Percentages of human ALL cells were monitored in the recipient’s peripheral blood (PB) and mice were treated with venetoclax, S63845, the combination of both, or vehicle for ten days (2 weeks, days 1-5) upon ALL manifestation (≥5% huALL cells in PB). An effective reduction of human ALL cells in PB was observed upon venetoclax and combination treatment in contrast to increasing leukemia cells showing no effect in control and S63845 treated animals (Fig. 7C). After treatment, animals were sacrificed and spleen weights (reflecting leukemia infiltration) and leukemia loads (huCD45 positive cells) were analyzed in spleen, bone marrow and central nervous system (Fig. 7D–G). Treatment with S63845 alone did not significantly affect leukemia loads and venetoclax reduced leukemia infiltration in spleens and the CNS. Importantly, the combination treatment resulted in markedly reduced leukemia loads in all compartments, indicating significant synergistic anti-leukemia activity of co-inhibition of BCL-2 and MCL-1 in vivo.

## Discussion

Alterations of the mitochondrial apoptosis pathway contribute to survival and treatment resistance of cancer cells, particularly of BCP-ALL [9, 29, 30], and therefore offer significant therapeutic targets. BH3-mimetics promote cell death by counteracting the anti-apoptotic function of BCL-2 family proteins, thereby activating MOMP followed by downstream apoptosis signaling [10, 31]. Previous studies have shown evidence for the efficacy of the BCL-2 inhibitor venetoclax in BCP-ALL cells [14–16, 32] and clinical data are being collected from case studies [33] and first clinical trials [34] (ClinicalTrials.gov: e.g., NCT03236857). Several MCL-1 inhibitors including S63845 [35], AMG176 [18] or AZD5591 [36] have been developed and are currently being investigated in preclinical models and first clinical trials (ClinicalTrials.gov: NCT02675452 (AMG176); NCT02979366, NCT03672695 (S63845); NCT03218683 (AZD5591)). S63845 binds with high affinity specifically to the BH3-binding groove of MCL-1 inducing downstream apoptosis signaling and has shown pre-clinical anti-tumor activity in different malignancies such as rhabdomyosarcoma [37], diffuse large B-cell lymphoma [38] and AML [39]. Also for BCL-XL, a small number of specific inhibitors including WEHI-539 [40], A1155463 [41] or A-1331852 [42] has lately been developed and is currently being tested and characterized in preclinical studies [43]. However, in the clinical application of these inhibitors, potential toxicity due to the role of BCL-XL in the human hematopoietic system needs to be considered in addition to its anti-tumor activity [44, 45].

In this study, we systematically analyzed the anti-leukemia activity of BH3-mimetics selectively targeting BCL-2 (venetoclax), BCL-XL (A-1331852) or MCL-1 (S63845) in BCP-ALL. Heterogeneous activity of the inhibitors was observed and effects were not associated with different leukemia characteristics like immunophenotype, recurrent genetic aberrations or treatment response criteria.

Insensitivity of ALL cells to these compounds was mediated by compensatory activation of other BCL-2 family proteins and could be overcome by combining these drugs. Investigating a series of seven cell lines and 27 PDX samples, our study did not reveal significant differences in the efficacy of the three inhibitors for cell death induction in BCP-ALL, emphasizing the relevance of BCL-XL and MCL-1 as therapeutic targets in addition to BCL-2. While several studies have provided clear evidence that CLL cells are mainly BCL-2 dependent [46, 47], it is less clear, which anti-apoptotic proteins are most important for cell survival in other malignancies. In line with our findings in ALL, a recent study also found heterogeneous sensitivity of diffuse large B-cell lymphoma cells to inhibitors of BCL-2, BCL-XL and MCL-1 [38]. In AML, however, another recent study identified a higher potency of S63845 for cell death induction in cell lines and primary cells as compared to venetoclax and A-1331852 [39]. A particular focus of recent leukemia research is the use of the combination of different BH3-mimetics inhibiting different BCL-2 family members. Recently, synergistic activity of BCL-2 and MCL-1 inhibition has been reported in AML PDX samples with acquired resistance to one of both inhibitors [48]. Further, synergistically reduced cell viability was demonstrated by dual and triple combinations of venetoclax, S63845 and A-1331852 in an in vitro CLL model [49]. Also, in T-ALL synergistic apoptosis induction of venetoclax and S63845 was found and the combination treatment was highly effective in a Myc-driven zebrafish T-ALL model [50]. In our study, co-targeting of BCL-2 with MCL-1 or BCL-XL induced synergistic cell death at nanomolar concentrations in most of the BCP-ALL samples. To further understand the mechanisms of the efficacy of this therapeutic approach, we analyzed the interplay of members of the BCL-2 family. Employing dynamic BH3 profiling, we found increased dependence on BCL-XL and MCL-1 in BCP-ALL cells upon venetoclax exposure. Venetoclax antagonizes the anti-apoptotic function of BCL-2 by binding to its BH3-binding domain, which leads to the release of pro-apoptotic proteins, most importantly BIM, and to the induction of apoptosis signaling [51, 52]. Assessing the interaction of BCL-2 family proteins using immunoprecipitation upon exposure of ALL cells to BH3-mimetics, we observed decreased binding of BIM to BCL-2 upon exposure to venetoclax, but compensational increased binding to MCL-1 (Fig. 7H), as described earlier in other cancer cell types [48, 49, 52, 53]. Conversely, exposure to S63845 disrupted BIM/MCL-1 with compensational increased BIM/BCL-2 binding, in accordance with a recent report analyzing multiple myeloma cells [54].

Remarkably, the combination of venetoclax and S63845 very efficiently impaired the binding of BCL-2 and MCL-1 to the apoptosis activator BIM, leading to synergistic cell death induction. Due to this observation, we focused our attention on the evaluation of BCL-2 and MCL-1 co-inhibition in patient-derived ALL xenograft samples. Synergistic activity was also present in PDX ALL samples using nanomolar concentrations of venetoclax and S63845. Most importantly, we also demonstrated enhanced anti-leukemia activity of venetoclax in combination with S63845 in PDX ALL in vivo, highlighting the efficacy of combining these drugs. However, although this therapeutic approach has effective anti-leukemia activity, studies are required to investigate possible side effects such as the occurrence of tumor lysis syndrome as recently described in an ALL PDX model [55] or effects on normal cells. Genetic studies have shown an important role of MCL-1 in cardiomyocytes [56] and for hematopoiesis [57, 58]. As the MCL-1 inhibitor S63845 has a higher affinity to human than to mouse MCL-1 [35], our xenograft study does not accurately address potential toxicity of the treatment. Therefore, the therapeutic window of MCL-1 inhibitors should be evaluated in other models including for example humanized *Mcl-1* mice [59].

Taken together, our study showed heterogeneous sensitivity of antagonizing BCL-2, BCL-XL and MCL-1 in BCP-ALL. We found an increased dependence of ALL cells on BCL-XL and MCL-1 upon exposure to venetoclax. Reciprocal binding of the apoptosis activator BIM to anti-apoptotic BCL-2 and MCL-1 was found upon treatment with venetoclax or S63845 and disrupted by combining both inhibitors, leading to proficient cell death signaling. Directly targeting this reciprocity by combined BCL-2 and MCL-1 inhibition showed efficient anti-leukemia activity, providing strong evidence for further evaluation of this combinatorial approach.

## Supplementary information

Supplementary data
