# Supplementary material for: Synergistic activity of combined inhibition of anti-apoptotic molecules in B-cell precursor ALL
Source: Leukemia. 2022 Jan 14;36(4):901–12. doi: 10.1038/s41375-021-01502-z (PMC8979822; doi:10.1038/s41375-021-01502-z)
Supplement: Supplementary file 1 — Supplementary data [file 41375_2021_1502_MOESM1_ESM.pdf]

Seyfried *et al*.

Synergistic Activity of combined inhibition of anti-apoptotic molecules in B-Cell Precursor ALL

Supplementary Methods:

BCP-ALL cell lines

RS4;11, KOPN-8, REH, EU-3, NALM-6 and RCH-ACV cells were purchased from DSMZ (Deutsche Sammlung für Mikroorganismen und Zellkulturen, Germany) and UoCB6 cells were provided by J. Rowley (USA). Lines were authenticated (STR profiling), mycoplasma contamination excluded (DAPI staining) and cultured in RPMI-1640 medium with 20% fetal bovine serum, 1% L-Glutamine and 1% Penicillin/Streptomycin (all Gibco) at 5% CO_2 and 37°C.

BCP-ALL patient-derived xenograft samples

Primary leukemia samples were collected from bone marrow or peripheral blood of pediatric patients with BCP-ALL after written informed consent of all patients and/or their representatives in accordance with the institution's ethical review board. Patient-derived xenograft samples were generated by intravenous transplantation of ALL cells into female NOD/SCID mice (NOD.CB17-Prkdcscid, Charles River) as previously described (1). Engraftment was monitored by staining peripheral blood and cell suspensions from organ compartments using anti-mCD45 (PE, #553081, BD), anti-huCD19 (APC, #555415, BD) and anti-huCD45 (PerCp, #345809, BD). Immunophenotyping was carried out according to standard protocols using an LSR-II flow cytometer (BD Biosciences). Genetic alterations were detected by Multiplex Ligation-dependent Probe Amplification (MLPA) and RT-PCR as previously described

(2). PDX cells were cultured in AIM-V (Gibco) at 5% CO_2 and 37°C. Animal experiments were approved by the responsible authorities (Regierungspräsidium Tübingen, Tierversuch Nr. 1260).

Cell viability assays

To determine half maximal effective concentration (EC_{50}) curves of BCP-ALL samples to BH3-mimetics, cells were exposed to ascending concentrations of venetoclax (#CT-A199, Chemietek), S63845 (#CT-S63845, Chemietek) and A-1331852 (#S7801, Selleckchem). Cell death rates were analyzed either according to propidium iodide (PI, #P3566, Invitrogen) positivity (cell lines) or forward/side scatter criteria (EC_{50} curves of PDX samples). Comparison of both methods yielded identical results of cell death detection as shown for RS4;11 (Supplementary Figure 1). EC_{50} values of seven cell lines were assessed after inhibitor exposure (2.5, 5, 25, 50, 250, 500 and 2500 nM) for 48h. EC_{50} values of 27 PDX samples were assessed after inhibitor exposure (1, 5, 10, 50, 100, 250, 500 nM, 1, 5 and 10 µM) for 24h.

Combination effects were analyzed by dose-response matrix analyses upon exposure to the three inhibitors (2.5, 5, 25, 50, 250, 500 and 2500 nM) for 48h (cell lines) or 24h (PDX samples) followed by cell death determination (PI staining). Compounds were dispensed in 384-well plates (automated Liquid Handling Station, Brand). Apoptosis of ALL cells was assessed by staining of Annexin V (APC, #550475, BD Pharmingen) and PI. All viability assays were measured on an Attune NxT Flow Cytometer with Autosampler (Thermo Fisher), data were analyzed using FlowJo 10.7.1 software.

Dynamic BH3 profiling

Dynamic BH3 profiling experiments were performed as described before (3, 4). In brief, cells were exposed to venetoclax for 2h (RS4;11, KOPN-8) or 4h (all others) and permeabilized (0.002% digitonin, #D5628, Sigma). Cells were exposed to pro-apoptotic BH3-peptides at increasing concentrations for 90 minutes, followed by fixation in formaldehyde for 10 minutes and incubation in a neutralization buffer for 10 min. Cells were stained with Dapi (#422801, Biolegend) and anti-cytochrome c antibody (#612308, Biolegend). Samples were measured using an Attune NxT Flow Cytometer (Thermo Fisher) and analyzed by FlowJo 10.7.1 software. Median fluorescence intensity (MFI) values of cytochrome c were determined and normalized to the MFIs of a negative (DMSO) and a positive control (Alamethicin, #BML-A150-0005, Enzo).

Intracellular protein staining

Cells were permeabilized (0.002% digitonin), fixed (formaldehyde), incubated in a neutralizing buffer, and stained with mouse anti-BCL-2 (Alexa Fluor 488, #59422, CST), rabbit anti-BCL-XL (Alexa Fluor 647, #86387, CST), rabbit anti-MCL-1 (Alexa Fluor 647, #78471, CST), mouse IgG1 Isotype Control (Alexa Fluor 488, #4878, CST) and rabbit IgG Isotype Control (Alexa Fluor 488, #4340, CST) in triplicates. Samples were measured (flow cytometry, Attune NxT, Thermo Fisher) and analyzed (FlowJo 10.7.1). MFIs were normalized to respective isotype controls.

Immunoprecipitation and immunoblotting

Cells were lysed (lysis buffer: 30 mM Tris-HCL, 150 mM NaCl, 2 mM KCl, 2 mM EDTA, 10% Glycerol, 1% Triton X, pH 7.4, supplemented with EDTA-free protease inhibitors

(Roche)) and protein concentrations were assessed (BCA protein assay, Thermo Fisher). Lysates were incubated with 88 ng of BIM Rabbit mAb (#2933, CST; overnight, 4°C) followed by Protein A Agarose beads (#9863S, CST; 2h, 4°C). Beads were washed 5x with lysis buffer and precipitates were subjected to western blot analyses. Lysates were mixed with Bolt^{TM} LDS sample buffer (Invitrogen) and Bolt^{TM} sample reducing agent (Invitrogen), heated (10 min, 70°C), separated in Bolt^{TM} 4-12% Bis-Tris Plus gels and transferred to a nitrocellulose membrane (iBlot Gel Transfer Device, Invitrogen). BCL-2 Mouse mAb (#15071, CST), BCL-XL Rb mAb (#2764, CST), MCL-1 Rb mAb (#94296, CST), BIM Rb mAb (#2933, CST), BID Rb Ab (#2002, CST), BAX Rb mAb (#5023, CST), BAK Rb mAb (#12105, CST), BAD Rb Ab (#9292, CST), BMF Rat mAb (ALX-804-343-C300, CST), PUMA Rb Ab (#P4743, Sigma), Noxa Mouse mAb (ALX-804-408-C100, Enzo), GAPDH Mouse mAb (#ADI-CSA-335-E, Enzo) and alpha-Tubulin Mouse mAb (#CP06, Calbiochem) were used as primary antibodies and mouse anti-rabbit IgG-HRP (sc-2357, Santa Cruz Biotechnology), mouse IgGκ BP-HRP (#sc-516102, Santa Cruz Biotechnology), goat anti-mouse IgG_1-HRP (#1070-05, Southern Biotech) or goat anti-rat IgG-HRP (#3050-05, Southern Biotech) as secondary antibodies. Immunoblots were developed using Western Lightning Plus-ECL (Perkin Elmer) and the Chemidoc Gel Imaging System (Bio-Rad). ImageJ software was used for densitometric quantification of immunoblots.

CRISPR/Cas9 mediated gene knockout

Two *MCL-1*-specific guide RNAs (5'-3' gRNA1: GCCTCGGCCCGGCGAGAGAT; gRNA2: GTAGCCAAAAGTCGCCCTCC) were designed based on the protocol by Ran et al. (5). These were cloned separately into the px335 backbone vector containing Cas9D10A. The vector was kindly provided by Feng Zhang (Addgene

plasmid: #42335). In order to exploit homology-directed repair for the selection of *MCL-1* knockout cells we designed an HDR-template consisting of two homology arms spanning 304nt upstream of gRNA1 and 541nt downstream of gRNA2 separated by EGFP and a BGH PolyA tail. Successful integration of the HDR-template resulted in in-frame integration of GFP at the *MCL-1* locus thereby allowing for GFP expression under the endogenous *MCL-1* promotor while abrogating *MCL-1* gene expression. BCP-ALL cells were co-transfected with Cas9D10A plasmids and the HDR-template using the NEON electroporation system (Invitrogen, USA) according to manufacturer's instructions. GFP+ cells were enriched two weeks after electroporation via flow cytometry and MCL-1 knockout was confirmed by western blot analysis.

***In vivo* treatment**

Mice were transplanted ($1x10^7$ ALL cells) and engraftment of human blasts was assessed in the peripheral blood by flow cytometry staining for mCD45, huCD19 and huCD45. Group sizes of five animals per condition had been estimated and upon engraftment of 5% human blasts in peripheral blood mice were randomly subdivided by consecutive numbering into groups of five animals per condition as determined before. Treatment was initiated with 25 mg/kg/d venetoclax orally (diluted in 10% Ethanol, 30% PEG, 60% Phosal 50 PG), 25 mg/kg/d S63845 intraperitoneally (diluted in 10% DMSO, 20% PEG, saline), combination or vehicle treatment (investigator non-blinded) five times per week for two consecutive weeks. After treatment mice were sacrificed and human leukemia burden was analyzed in recipient mice. Moribund animals with detection of strain-specific murine thymoma (mCD45 positive, huCD45 negative murine cells) were excluded from analysis, as specified before the experiment.

Statistical analysis

Statistical analyses were performed with GraphPad Prism 9 software and Microsoft Excel. Data obtained from three independent experiments carried out in triplicates (cell lines) or from one experiment performed in triplicate (PDX samples) were analyzed assuming equal variances. 3D scatter plots of EC_{50} values were generated using Plotly software (6). Synergyfinder was used to analyze combination effects of the inhibitors and to calculate synergy scores based on the Bliss independence model (7-9).

Supplementary Figure 1: Comparison of strategies to assess cell death in ALL cells

(A) Forward/side scatter analysis was investigated in parallel to propidium iodide (PI) staining (exemplary shown for RS4;11), showing similar results of cell death detection in ALL cell lines. **(B)** Spearman correlation of cell death assessed by forward/side scatter criteria and PI staining. Spearman correlation; r, correlation coefficient; p, significance.

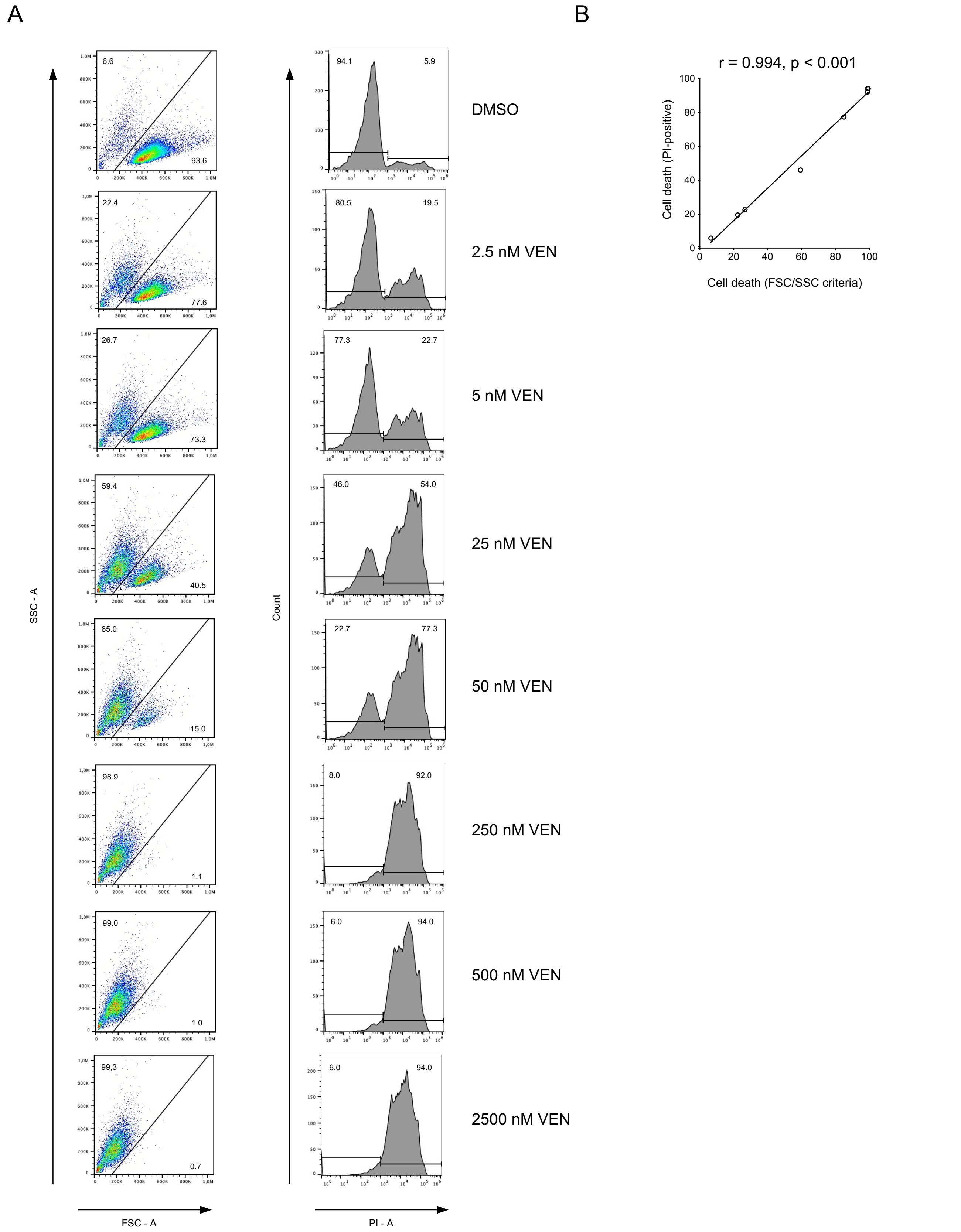

Supplementary Figure 2: EC_{50} values of BCP-ALL cell lines

B-cell precursor acute lymphoblastic leukemia (BCP-ALL) cell lines were exposed to venetoclax, S63845 or A-1331852 for 48 hours followed by assessment of cell death determined by propidium iodide staining. Half maximal effective concentrations (EC_{50}) were calculated for the three compounds. Sensitivities of the compounds are color-coded as shown in the legend.

	EC_{50} [nM]		
**ID**	**Venetoclax**	**S63845**	**A-1331852**
RS4;11	32	325	1195
KOPN-8	46	10	390
UoCB6	216	37	441
REH	75	55	1520
EU-3	157	92	930
RCH-ACV	2362	233	544
NALM-6	31302	2137	138
Median	157	92	544

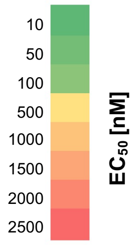

Supplementary Figure 3: Characteristics of BCP-ALL cell lines

Association of characteristics of BCP-ALL cell lines (10-13) with their sensitivities towards BH3-mimetics. nk, not known.

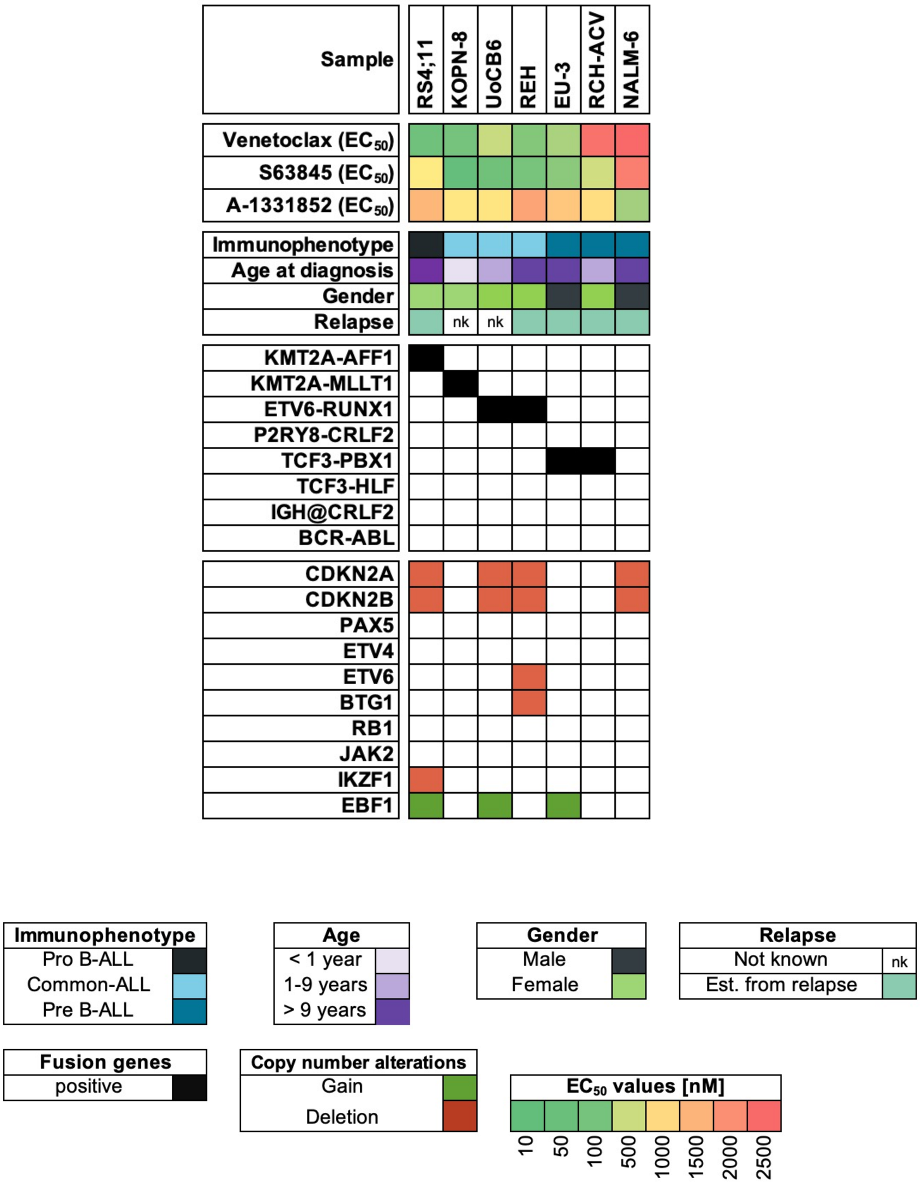

Supplementary Figure 4: EC_{50} values of BCP-ALL patient-derived xenograft samples

Cell death was analyzed after exposure of cell lines to venetoclax, S63845 or A-1331852 for 24 hours. Half maximal effective concentrations (EC_{50}) were calculated for the three compounds. Sensitivities of the compounds are color-coded as shown in the legend.

	EC_{50} [nM]		
**ID**	**Venetoclax**	**S63845**	**A-1331852**
PDX-21	18	190	325
PDX-19	27	49	14
PDX-22	35	74	6
PDX-08	54	204	176
PDX-10	102	84	16
PDX-20	111	1052	622
PDX-15	113	273	773
PDX-07	122	546	6336
PDX-13	136	221	135
PDX-26	170	634	1061
PDX-14	198	341	73
PDX-24	249	944	2313
PDX-18	439	169	7552
PDX-25	667	693	50
PDX-02	1028	425	1883
PDX-11	1100	1430	1211
PDX-16	1444	200	140
PDX-17	1637	2285	1243
PDX-06	1800	409	156
PDX-03	2967	6827	4069
PDX-01	3247	89	10
PDX-04	6209	3107	1166
PDX-23	7195	11584	7413
PDX-09	7677	1107	10
PDX-27	13694	526	2064
PDX-05	13883	1297	13892
PDX-12	34929	11038	1756
Median	667	526	773

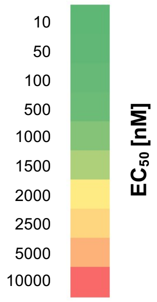

Supplementary Figure 5: Patient-derived xenograft samples and corresponding characteristics ordered according to BH3-mimetic sensitivity

5A: Venetoclax

PDX samples are sorted according to venetoclax EC_{50} values from left (sensitive) to right (insensitive).

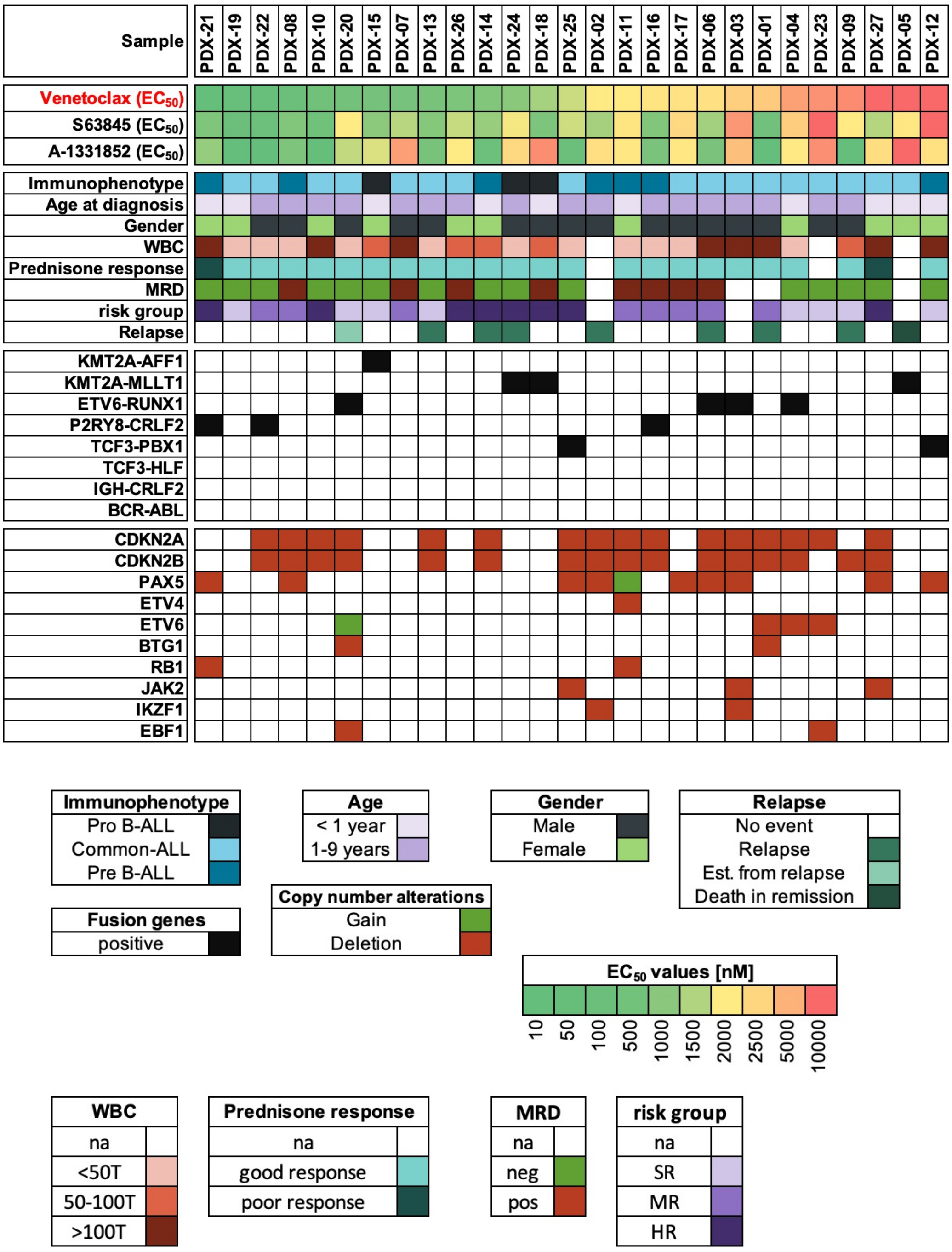

5B: S63845

PDX samples are sorted according to S63845 EC_{50} values from left (sensitive) to right (insensitive).

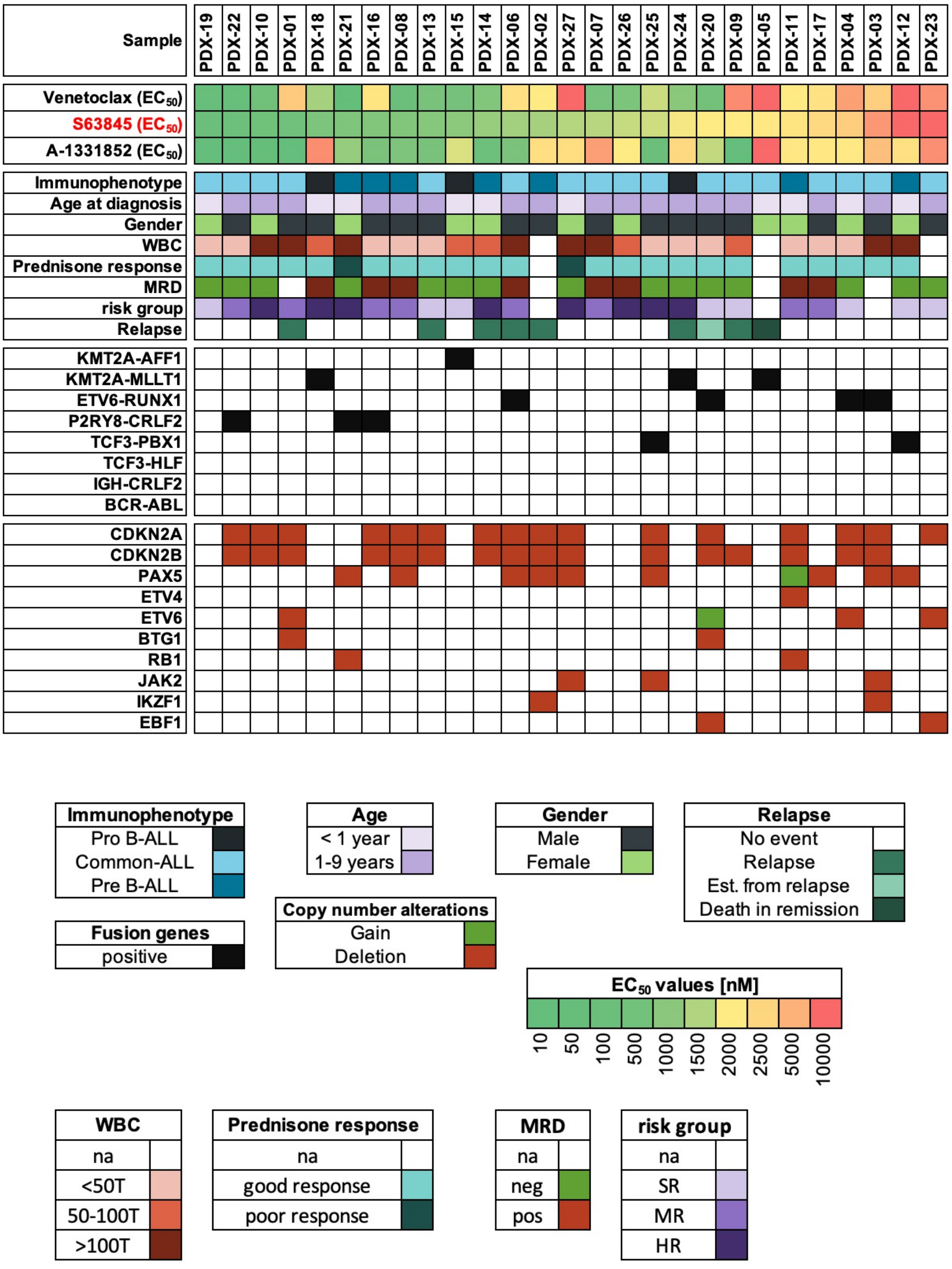

5C: A-1331852

PDX samples are sorted according to A-1331852 EC_{50} values from left (sensitive) to right (insensitive).

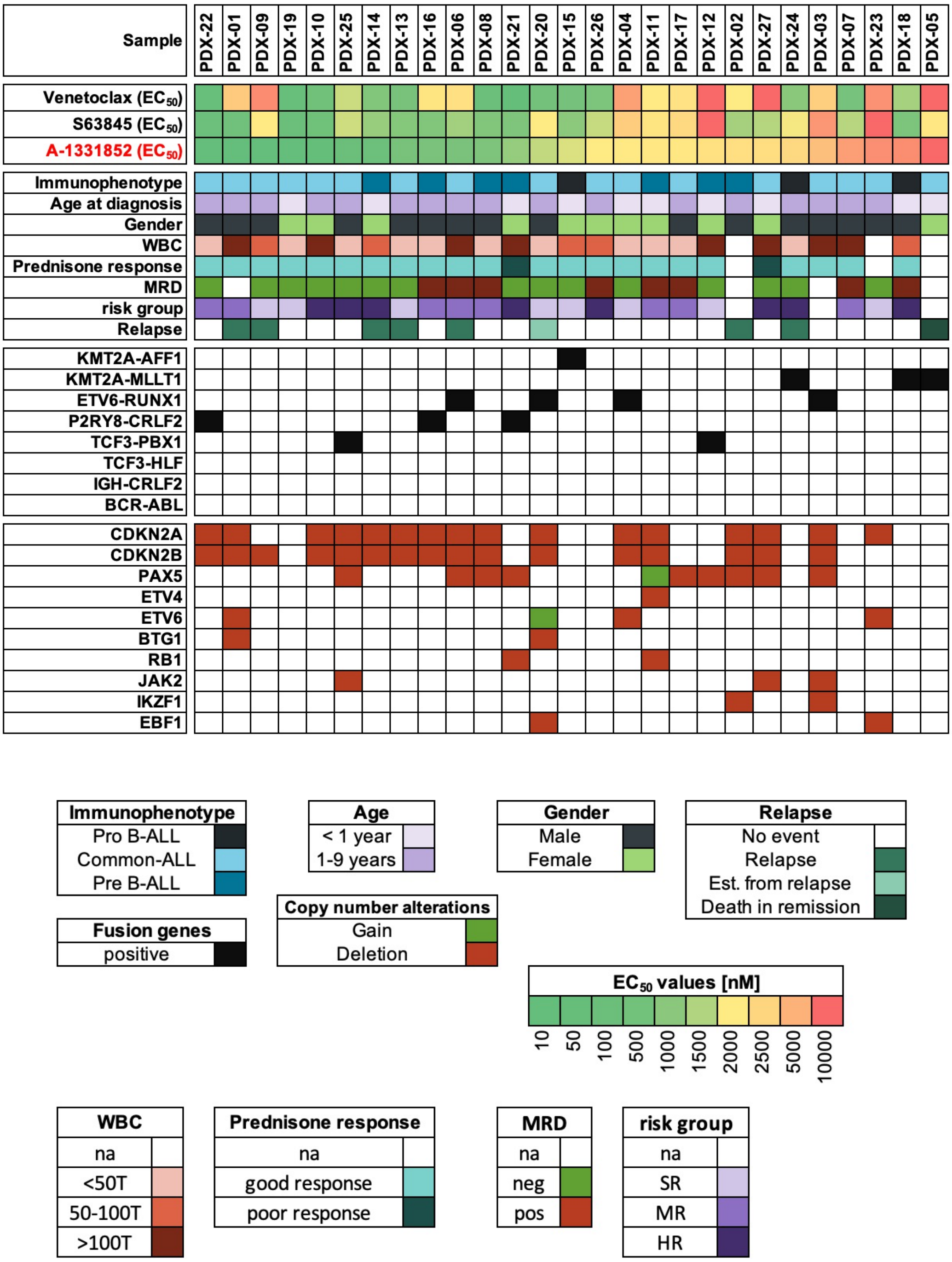

5D: Comparison of PDX samples with sensitivity (N=20) and insensitivity (N=7) to all three BH3-mimetics

Samples were divided into two groups showing sensitivity (EC_{50} < 1 µM) to at least one of the three inhibitors (left, n=20) or insensitivity to all three inhibitors (EC_{50} ≥ 1 µM) (right, n=7).

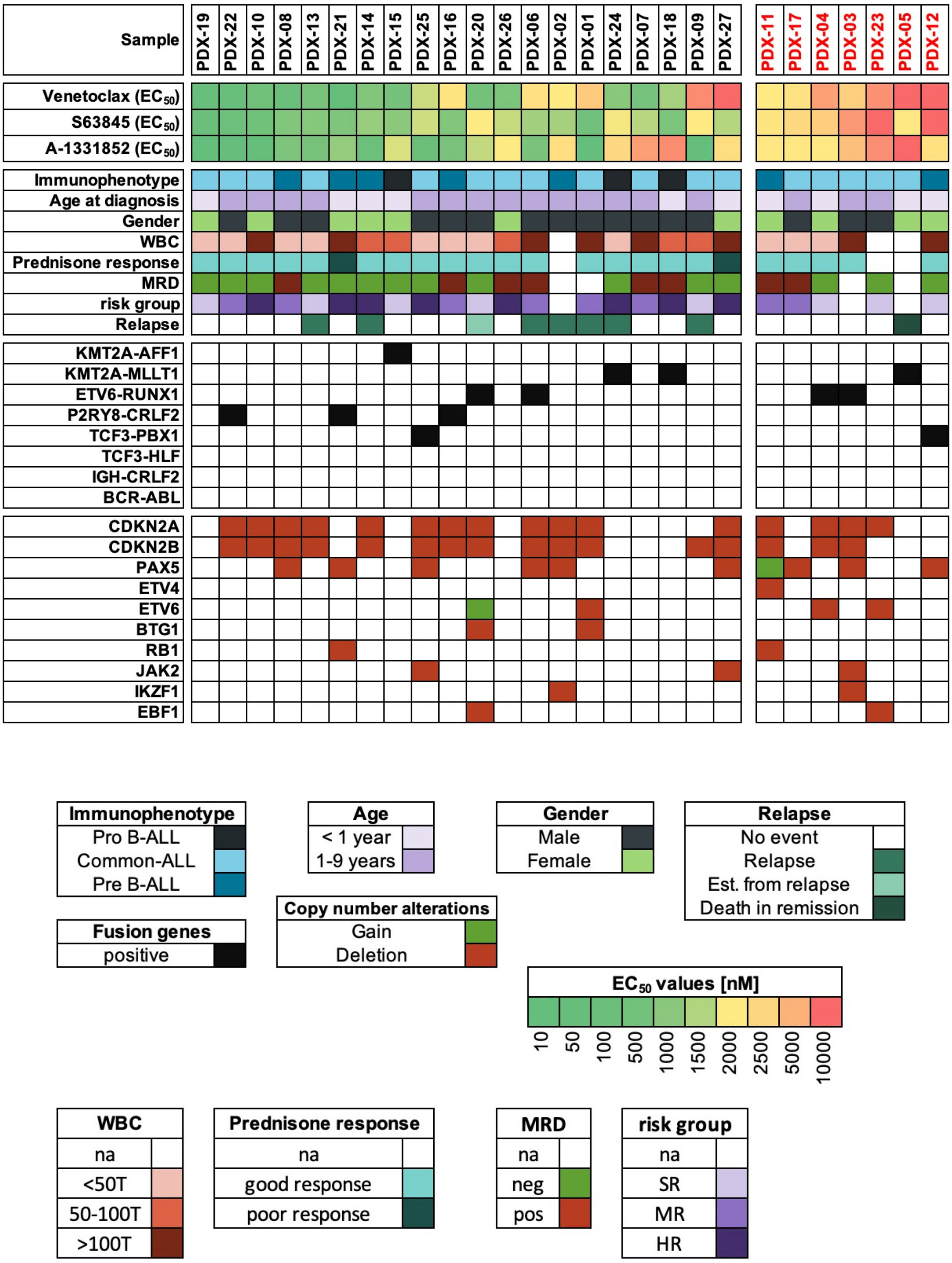

Supplementary Figure 6: Protein expression levels of BCL-2, BCL-XL and MCL-1

Basal protein levels of the three target proteins (BCL-2, MCL-1 and BCL-XL) of venetoclax, S63845 and A-1331854 were determined by FACS staining. **(A)** Representative FACS plots are shown for PDX-18 (high BCL-2, low BCL-XL and MCL-1) and PDX-09 (low BCL-2, high BCL-XL and MCL-1). Filled histograms represent the proteins indicated and dotted lines are the isotype controls. **(B)** Calculated mean fluorescence intensity ratios (MFIR) of BCL-2 (Alexa Fluor 488), BCL-XL (Alexa Fluor 647) and MCL-1 (Alexa Fluor 647) normalized to their isotype controls are shown for all samples. All values shown are mean values from triplicates.

A

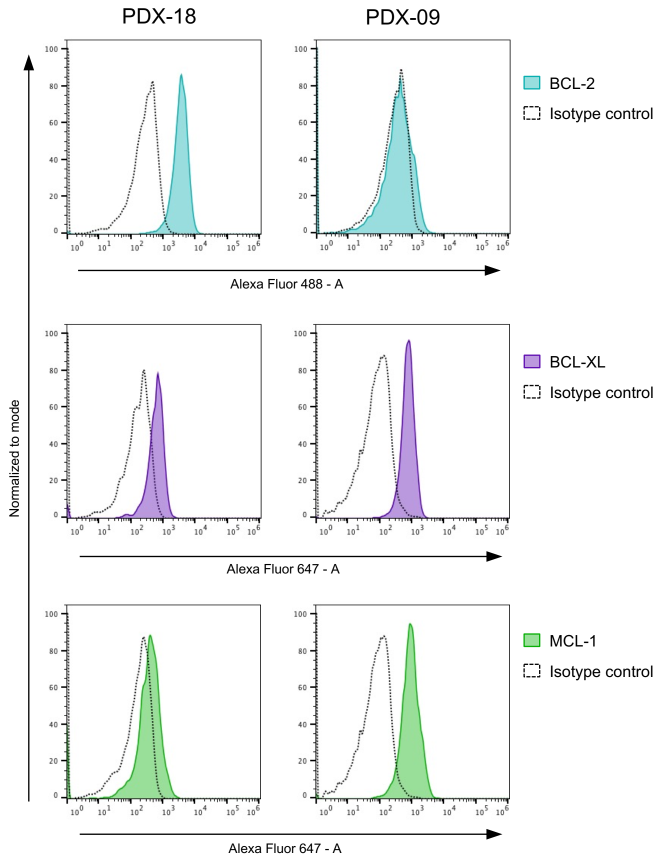

B

	Protein level [MFIR]		
**ID**	**BCL-2**	**BCL-XL**	**MCL-1**
PDX-21	na	na	na
PDX-19	6.01	3.12	2.11
PDX-22	na	na	na
PDX-08	17.99	7.15	2.54
PDX-10	9.61	6.06	2.62
PDX-20	8.51	4.15	2.19
PDX-15	12.11	7.14	5.29
PDX-07	5.50	7.48	5.55
PDX-13	12.64	5.29	2.77
PDX-26	13.64	3.26	2.07
PDX-14	8.23	4.06	1.84
PDX-24	11.66	2.92	2.89
PDX-18	17.58	2.13	1.15
PDX-25	8.98	4.64	2.77
PDX-02	na	na	na
PDX-11	8.03	8.51	5.20
PDX-16	7.62	5.16	4.81
PDX-17	10.84	7.60	7.28
PDX-06	9.23	9.20	5.80
PDX-03	8.50	5.62	4.52
PDX-01	2.48	7.84	5.88
PDX-04	8.80	4.05	2.38
PDX-23	13.22	5.71	5.28
PDX-09	1.51	17.15	12.62
PDX-27	5.16	4.29	4.32
PDX-05	4.49	3.43	3.49
PDX-12	2.77	6.19	6.22

Supplementary Figure 7: Protein expression levels of pro-apoptotic regulators are not associated with inhibitor sensitivities

(A) Basal protein levels of the pro-apoptotic regulator proteins (BIM, BID, BAX, BAK, BAD, BMF, PUMA and Noxa) in the seven BCP-ALL cell lines determined by western blot analysis. Sensitivities of the compounds are color-coded as shown in the legend.

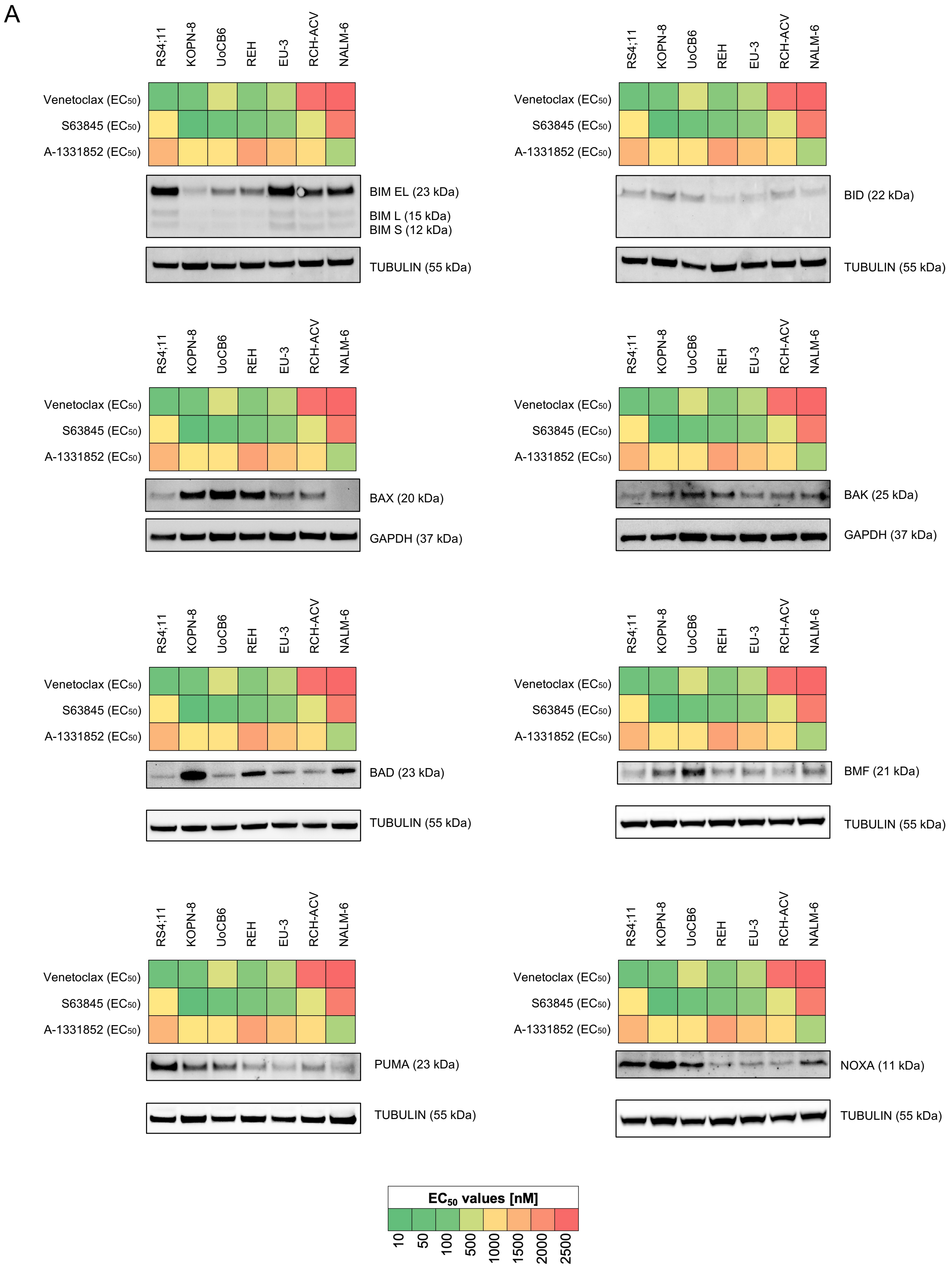

Supplementary Figure 7: Protein expression levels of pro-apoptotic regulators are not associated with inhibitor sensitivities

(B) Expression levels of BIM, BID, BAX, BAK, BAD, BMF, PUMA and Noxa were quantified by densitometry (ImageJ) and correlated to the EC_{50} values of the respective inhibitors.

B

	**Venetoclax**		**S63845**		**A-1331852**	
	r_s	p	r_s	p	r_s	p
**BIM**	0.071	0.906	0.750	0.066	0.321	0.498
**BID**	0.250	0.595	-0.536	0.236	-0.607	0.167
**BAX**	-0.214	0.662	-0.893	0.012	0.214	0.662
**BAK**	-0.071	0.906	-0.500	0.267	-0.500	0.200
**BAD**	-0.464	0.302	-0.250	0.595	-0.071	0.906
**BMF**	0.643	0.139	-0.250	0.595	-0.857	0.024
**PUMA**	-0.357	0.444	-0.071	0.783	-0.143	0.783
**NOXA**	-0.464	0.302	-0.036	0.963	-0.321	0.498

Spearman correlation of EC_{50} values of BH3-mimetics with the protein levels indicated. N=7 cell lines; p, significance; Bonferroni-adjusted significance level: $P \leq 0.05/24 = P \leq 0.002$.

Supplementary Figure 8: Alternate binding of BIM upon BCL-2 and MCL-1 inhibition

Independent second and third BIM co-immunoprecipitation experiments for RS4;11 **(A)**, NALM-6 **(B)** and RCH-ACV **(C, D)** showing co-precipitated/BIM-bound BCL-2 or MCL-1 upon venetoclax or S63845 exposure at the concentrations indicated for four hours. See also Figure 3.

A

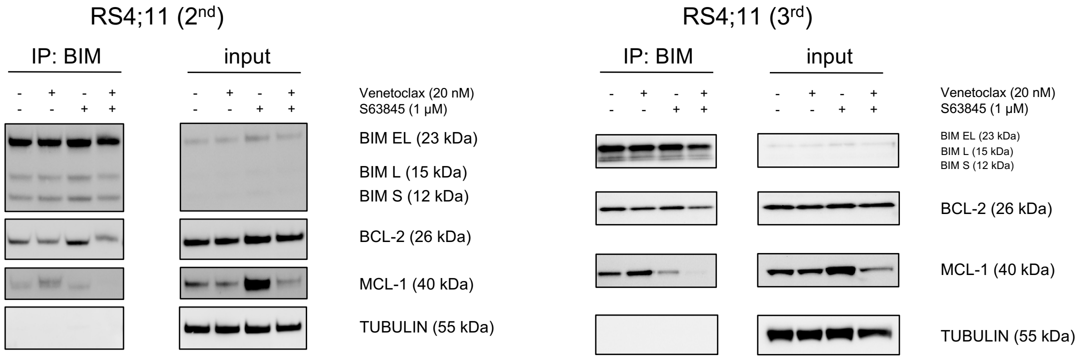

B

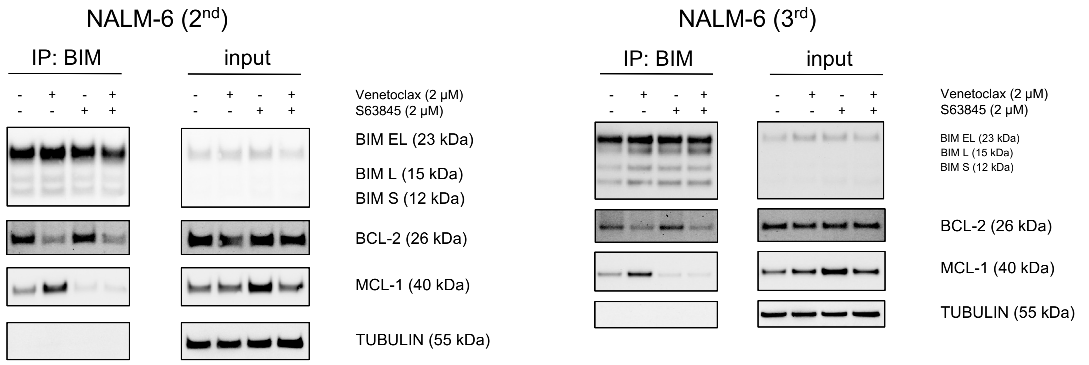

C

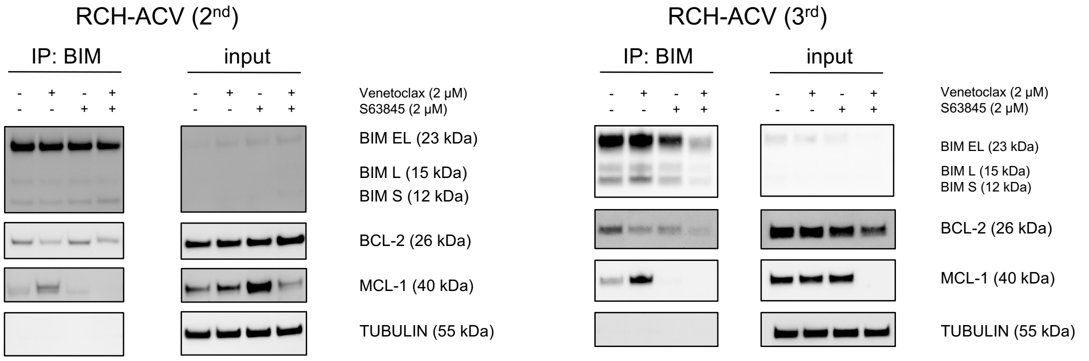

D

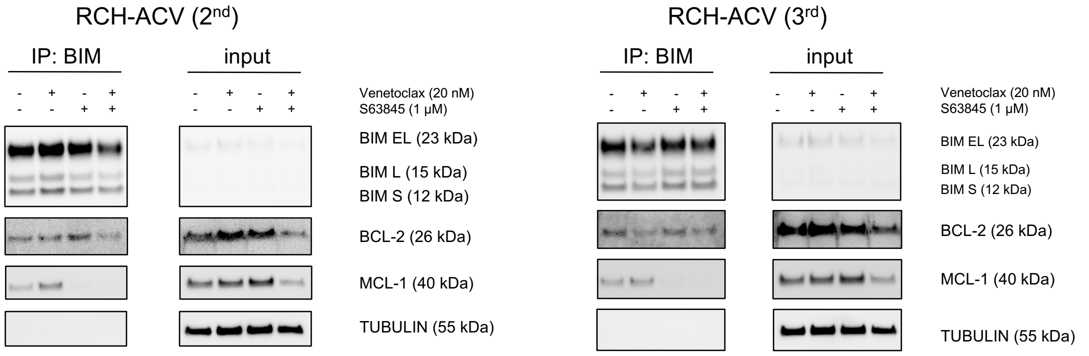

Supplementary Figure 9: Alternate binding of BIM upon BCL-2 and BCL-XL inhibition

(A) BIM co-precipitation in NALM-6 cells. Detection of co-precipitated/BIM-bound BCL-2 and BCL-XL (upper band corresponding to BCL-XL) upon exposure to venetoclax and/or A-1331852 (both 2 µM, four hours). **(B)** Densitometric quantification of co-precipitated BCL-2 or BCL-XL (upper band) in the respective condition relative to no inhibitor control summarizing the three independent experiments. Unpaired two-tailed Student's T-test; significance ***, p<.001; **, p<.01; *, p<.05; ns, not significant.

A

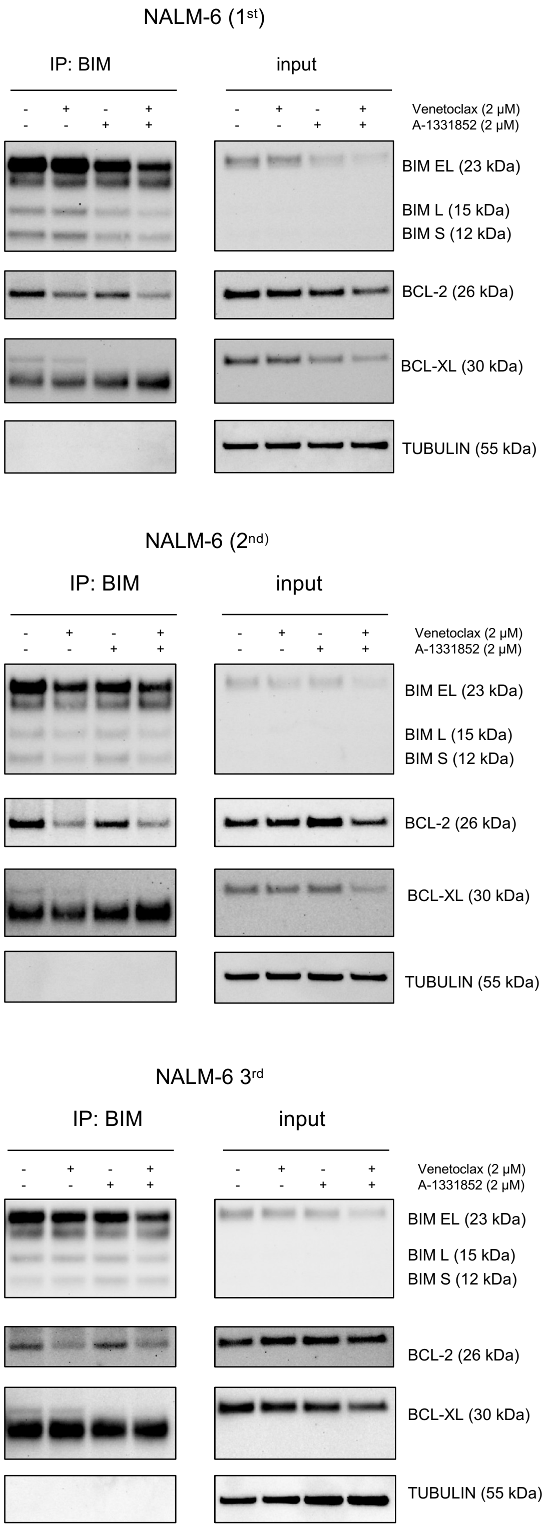

B

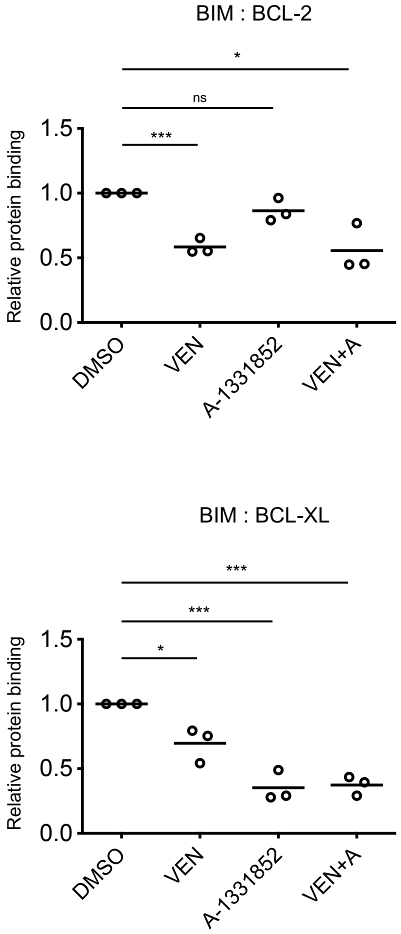

Supplementary Figure 10: BIM shuttling upon inhibitor treatment is a rapid process

In order to get insight into the chronological sequence of increased BIM protein binding to MCL-1, RS4;11 cells were exposed to 20 nM venetoclax for 1, 2 and 4 hours followed by immunoprecipitation (IP) analysis of BIM. The immunoprecipitation lanes show increased binding of BIM and MCL-1 at all time points investigated. Input lanes show whole protein lysates.

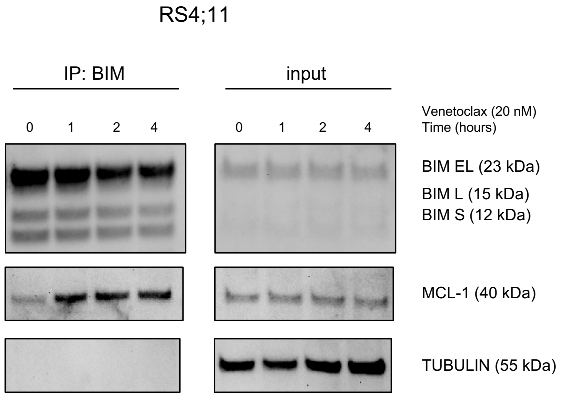

Supplementary Figure 11: Annexin V / PI staining

Exemplary plots showing Annexin V and PI staining in RS4;11 cells following exposure for 48 hours to 100 nM S63845 and/or 10 nM venetoclax or 100 nM A-1331852 and/or 10 nM venetoclax. Quantification of viable cells (Annexin V-/PI-), early apoptotic cells (Annexin V+/PI-), late apoptotic cells (Annexin V+/PI+) and necrotic cells (Annexin V-/PI+) was performed from three independent experiments in triplicates (shown in Figure 5D and 6D).

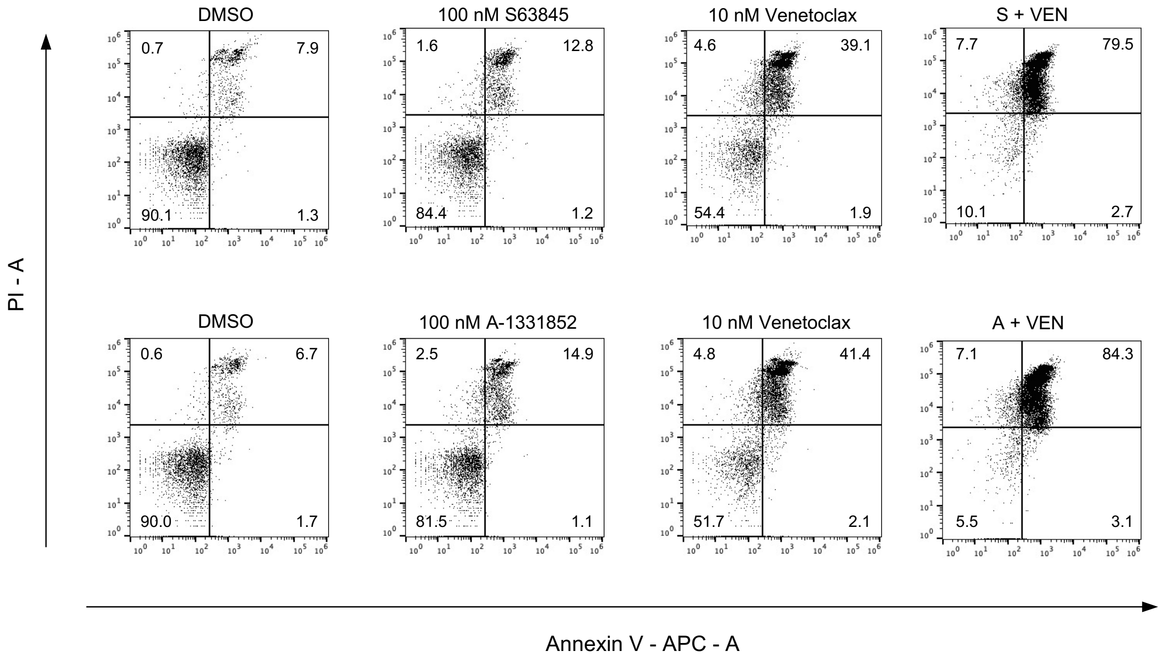

Supplementary Figure 12: Combination effects of venetoclax and A-1331852 in ALL patient-derived xenograft samples

Dose-response matrix analyses of cell death (propidium iodide staining) following exposure for 24 hours to 2.5, 5, 25, 50, 250, 500 and 2500 nM venetoclax and/or A-1331852 are shown. δ-scores were determined by synergyfinder and synergistic effects are shown in red, additive effects in white and antagonistic effects in green. The Bliss synergy score indicates the average synergy score over the dose-response matrix.

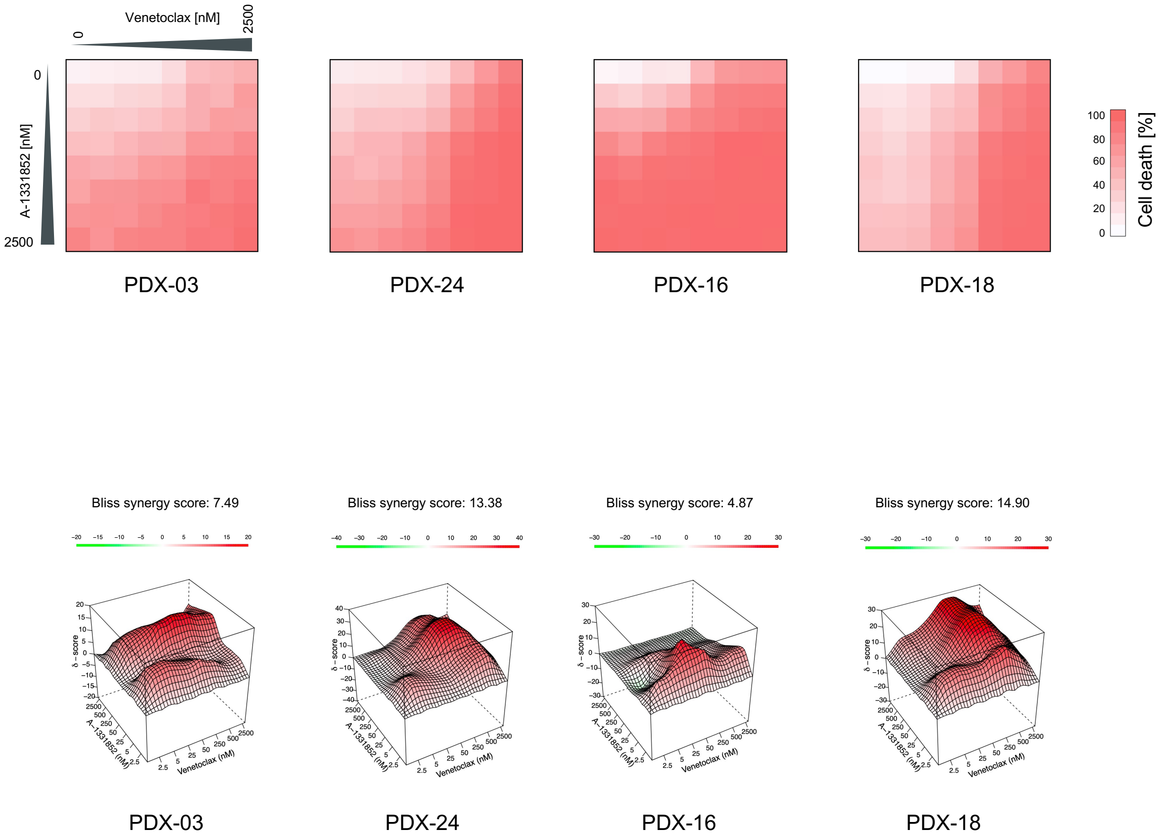

Supplementary References:

1. Meyer LH, Eckhoff SM, Queudeville M, Kraus JM, Giordan M, Stursberg J*, et al.* Early relapse in ALL is identified by time to leukemia in NOD/SCID mice and is characterized by a gene signature involving survival pathways. *Cancer cell* 2011 Feb 15; **19**(2)**:** 206-217.
2. Boldrin E, Gaffo E, Niedermayer A, Boer JM, Zimmermann M, Weichenhan D*, et al.* MicroRNA-497/195 is tumor-suppressive and cooperates with CDKN2A/B in pediatric acute lymphoblastic leukemia. *Blood* 2021 Jun 7.
3. Ryan J, Montero J, Rocco J, Letai A. iBH3: simple, fixable BH3 profiling to determine apoptotic priming in primary tissue by flow cytometry. *Biological chemistry* 2016 Jul 01; **397**(7)**:** 671-678.
4. Montero J, Sarosiek KA, DeAngelo JD, Maertens O, Ryan J, Ercan D*, et al.* Drug-induced death signaling strategy rapidly predicts cancer response to chemotherapy. *Cell* 2015 Feb 26; **160**(5)**:** 977-989.
5. Ran FA, Hsu PD, Wright J, Agarwala V, Scott DA, Zhang F. Genome engineering using the CRISPR-Cas9 system. *Nature protocols* 2013 Nov; **8**(11)**:** 2281-2308.
6. Inc. PT. Collaborative data science. Montréal: Plotly Technologies Inc.; 2015.
7. Foucquier J, Guedj M. Analysis of drug combinations: current methodological landscape. *Pharmacol Res Perspect* 2015 Jun; **3**(3)**:** e00149.
8. Zhao W, Sachsenmeier K, Zhang L, Sult E, Hollingsworth RE, Yang H. A New Bliss Independence Model to Analyze Drug Combination Data. *J Biomol Screen* 2014 Jun; **19**(5)**:** 817-821.

9. Ianevski A, Giri AK, Aittokallio T. SynergyFinder 2.0: visual analytics of multi-drug combination synergies. *Nucleic Acids Res* 2020 Jul 2; **48**(W1)**:** W488-W493.

10. Drexler HG. Guide to Leukemia- Lymphoma Cell Lines, 2nd Edition. *Braunschweig*2010.

11. Stong RC, Korsmeyer SJ, Parkin JL, Arthur DC, Kersey JH. Human acute leukemia cell line with the t(4;11) chromosomal rearrangement exhibits B lineage and monocytic characteristics. *Blood* 1985 Jan; **65**(1)**:** 21-31.

12. Kim DH, Moldwin RL, Vignon C, Bohlander SK, Suto Y, Giordano L*, et al.* TEL-AML1 translocations with TEL and CDKN2 inactivation in acute lymphoblastic leukemia cell lines. *Blood* 1996 Aug 1; **88**(3)**:** 785-794.

13. Duque-Afonso J, Lin CH, Han K, Wei MC, Feng J, Kurzer JH*, et al.* E2A-PBX1 Remodels Oncogenic Signaling Networks in B-cell Precursor Acute Lymphoid Leukemia. *Cancer research* 2016 Dec 1; **76**(23)**:** 6937-6949.
